# Cost-effectiveness analysis of the covered endovascular reconstruction of the aortic bifurcation versus kissing stents and open surgical repair for the treatment of aorto-iliac occlusive disease

**DOI:** 10.1007/s10198-025-01792-5

**Published:** 2025-07-30

**Authors:** Xavier G. L. V. Pouwels, Suzanne Holewijn, Daphne van der Veen, Mauricio Gonzalez-Urquijo, Michel M. P. J. Reijnen, Hendrik Koffijberg

**Affiliations:** 1 Section of Health Technology and Services Research, TechMed Centre, Faculty of Behavioural, Management, and Social Sciences, University of Twente, Enschede, The Netherlands; 2 Department of Surgery, Rijnstate, Arnhem, The Netherlands; 3 Tecnologico de Monterrey, School of Medicine and Health Sciences, Monterrey, Mexico; 4 Multi-Modality Medical Imaging Group, Technical Medical Centre, Faculty of Science and Technology, University of Twente, Enschede, The Netherlands

**Keywords:** Cost-effectiveness analysis, Economic model, Endovascular procedures, Arterial occlusive diseases, Quality-adjusted life years

## Abstract

**Objective:**

To assess the cost effectiveness of the Covered Endovascular Reconstruction of the Aortic Bifurcation (CERAB) versus kissing stents (KS) and open surgical repair (OSR) for treating extensive aorto-iliac occlusive disease (AIOD).

**Methods:**

A decision tree followed by a health state transition model was developed to simulate changes in Rutherford status and the occurrence of reinterventions, amputation, and death. A Dutch health care perspective and a five-year time horizon were used. Model inputs were estimated using non-randomised data of individuals who underwent a CERAB or a KS procedure and literature. The total number of reinterventions, life years, quality-adjusted life years (QALYs) and health care costs per strategy were calculated as well as the incremental costs and QALYs between strategies, and corresponding incremental cost-effectiveness ratios (ICERs).

**Results:**

OSR resulted in the lowest survival due to a higher peri-operative probability of death. OSR resulted in a lower probability of reinterventions (6%, 95% Confidence Interval (CI): 1-15%) than CERAB (17%, 95%CI: 11-27%) and KS (29%, 95%CI: 17-46%). CERAB dominated OSR since it led to 0.032 (95%CI: -0.038-0.082) incremental QALYs and €-11,466 (95%CI: €-18,934-€-3,415) incremental costs versus OSR. CERAB led to 0.048 (95%CI: 0.011–0.109) incremental QALYs, €5,324 (95%CI: €2,938-€10,397) incremental costs, and an ICER of €110,201 per QALY versus KS.

**Conclusions:**

CERAB dominated OSR and resulted in the highest health benefits and costs but does not seem to be cost effective versus KS for treating AIOD. Performing a randomised comparison of these treatment modalities is essential to confirm these findings.

**Supplementary Information:**

The online version contains supplementary material available at 10.1007/s10198-025-01792-5.

## Introduction

Aorto-iliac occlusive disease (AIOD) can be treated with endovascular techniques or by open surgical repair (OSR). Guidelines still favour performing OSR in medically fit individuals with extensive AIOD due to the higher long-term patency rates of OSR compared to endovascular techniques [1, 2], but there is also room for endovascular strategies in sites with sufficient experience [3]. A potential explanation for lower patency rates after endovascular treatment may be a suboptimal geometry obtained with kissing stents (KS) [4], which may lead to unfavorable flow conditions and subsequent risk on thrombosis and restenosis. In 2009, the Covered Endovascular Reconstruction of the Aortic Bifurcation (CERAB) was introduced for treating AIOD [5]. CERAB aimed to achieve a better anatomical and physiological reconstruction, and thereby may improve long-term outcomes obtained with endovascular reconstructions. A recent meta-analysis of mostly observational studies on extensive AIOD showed that CERAB reached higher three-year patency rates than standard endovascular treatment but still had lower three-year patency rates than OSR [1]. There is however no randomised controlled trial comparing CERAB to other endovascular revascularisations and/or OSR.

In light of rising healthcare costs and the (upcoming) shortage of medical staff [6], it is increasingly important to use scarce healthcare resources most efficiently and to assess economic outcomes besides health outcomes. Previous studies have shown that OSR leads to more peri-procedural mortality, longer hospital stays and higher inpatient costs compared with endovascular revascularisation [7, 8]. These studies however focused only on hospital costs of endovascular revascularisation in general and OSR, they did not consider CERAB, and they did not consider the long-term consequences of these treatments, such as reinterventions. These comparisons are thus insufficient to identify the most cost-effective procedure for treating AIOD.

Due to this lack of formal health economic (HE) evaluation of CERAB versus other endovascular revascularisation modalities and OSR [1, 4], it is still unclear whether the shorter operation time and hospital stay and lower 30-day mortality of CERAB and endovascular revascularisations outweigh the long-term benefits of OSR, such as less reinterventions. Currently, the EVOCC trial (UK National Institute for Health and Care Research award: NIHR151230) is investigating the clinical and cost-effectiveness of OSR versus any endovascular treatment in the United Kingdom, which will address these questions. However, the results of the EVOCC trial are not expected before the end of 2028, leaving uncertainty concerning which of these treatments is most cost effective in this patient population.

Therefore, the aim of this study was to explore the cost effectiveness of CERAB versus both KS and OSR for treating extensive AIOD with predominantly Trans-Atlantic Inter-Society Consensus (TASC) II C and D lesions.

## Methods

### Decision problem and setting

HE evaluations are systematic and transparent comparisons of the health outcomes and costs of different treatment strategies [9]. This HE evaluation aimed at assessing the cost-effectiveness of CERAB versus KS and OSR in individuals who were treated electively for AIOD. The mean age of the simulated population was 61.6 years old and composed of 50% of women. Simulated individuals predominantly suffered from TASC II C (8%) and D (82%) lesions. These population characteristics were based on the datasets we have used to inform this HE evaluation.

The evaluation was performed from a Dutch healthcare perspective and a time horizon of 5 years, which was deemed sufficient to estimate the difference in costs and effects between all strategies. The outcomes of interests were the number of reinterventions in each strategy, life years (LYs), quality-adjusted life years (QALYs), and health care costs. Annual discount rates of 1.5% and 4% were used for health effects and costs respectively [10]. No Institutional Review Board approval was obtained for this study.

### Model structure

**Fig. 1:**
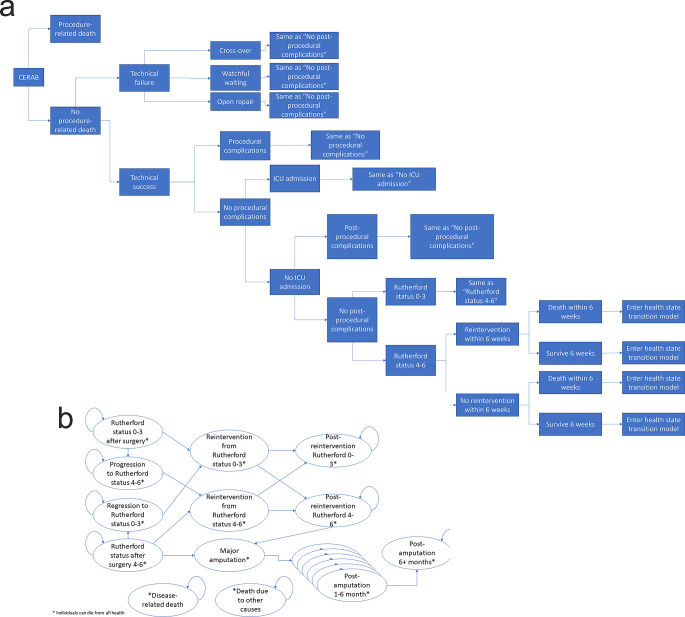
Model structure. **A**: Decision tree. **B**: Health state transition model

A combination of a decision tree and health state transition model (HSTM) was developed to assess the cost-effectiveness of CERAB versus KS and OSR. The decision tree simulated the trajectory of individuals undergoing any of the treatment for a period of six weeks. Individuals could experience (post-)procedural complications, a reintervention, die, and be admitted to the intensive care unit (ICU) during these six weeks (Fig. 1A). Procedural complications led to an increase in surgery time, while post-procedural complications led to an increase in hospitalisation time. The Rutherford status of individuals after surgery and the occurrence of a reintervention within these six weeks determined in which health states individuals entered the HSTM.

The HSTM simulated disease progression using the Rutherford classification and the occurrence of reinterventions and amputation after these initial 6 weeks until 5 years. The HSTM contained 18 health states (Fig. 1B). In both the decision tree and HTSM, Rutherford statuses 0–3 (R0-3) and Rutherford statuses 4–6 (R4-6) were combined in a single health state.

### Transition probabilities and relative effectiveness

Transition probabilities of the HE model were estimated using individual-level data from two datasets containing information on individuals who underwent a CERAB and KS (available to the authors), and literature. The CERAB dataset was collected at one single high volume site in the Netherlands [11], and contained (a) individuals’ characteristics pre-, peri-, and post-procedure: technical success, Rutherford statuses before and after the intervention, duration of the procedure, and length of hospital stay, and (b) 5-year follow-up data concerning individuals’ Rutherford status, patency, and the occurrence of reintervention [11, 12]. The KS dataset was previously collected from multiple observational studies to perform an individual-level meta-analysis and contained information on individuals’ characteristics pre-, peri- and post-procedure and the month until patency loss and the occurrence of reintervention [13].

Missing data in the two datasets were imputed using multiple imputation resulting in ten complete datasets [Appendix]. The CERAB multiply-imputed datasets were used to estimate the model inputs of the decision tree. The pathways’ probabilities were assumed equal between CERAB and KS, since both are endovascular procedures. The pathways’ probabilities for OSR were obtained from literature [2, 14].

The probabilities to progress from R0-3 to R4-6 and to regress from R4-6 to R0-3 were estimated using parametric survival models fitted to the CERAB data, and were assumed equal across all strategies. The best fitting model was selected using the Akaike Information Criterion since these probabilities were not extrapolated beyond the follow-up time.

The probabilities of reintervention in the CERAB and KS strategies were estimated using all multiply-imputed datasets of CERAB and KS. To adjust for differences in baseline characteristics between the CERAB and KS groups, we matched the KS group to the CERAB group using a genetic matching algorithm [15] [Appendix]. After matching, survival analyses were used to estimate the probability of reintervention in the CERAB and KS strategies. To estimate the probability of reintervention in the OSR strategy, a hazard ratio for the occurrence of reintervention in the OSR strategy versus CERAB was estimated using a matching-adjusted indirect treatment comparison (MAIC) [16] [Appendix]. The probability of amputation, assumed equal over all strategies, was obtained from literature [17]. Age and gender-specific general population mortality rates were used to estimate the probability of death due to other causes [18].

Since probabilities relating to disease progression were assumed equal between the strategies, differences in health outcomes and costs between the strategies were caused by the different probabilities of experiencing a reintervention of the strategies.

### Health effects

The health effects estimated in the current health economic model were the occurrence of reinterventions, LYs, and QALYs.

To estimate QALYs in the decision tree, the baseline utility value in all strategies was assumed equal and was calculated based on the pre-surgery distribution of Rutherford status among individuals undergoing a CERAB. This baseline utility value was applied for the duration of the hospital stay at the regular ward for individuals who did not experience a post-procedural complication. The disutility of a reintervention was applied to the hospital stay at the regular ward for individuals who experienced a post-procedural complication and for individuals who were admitted to the intensive care unit. For the remaining of the decision tree, utility values were determined based on the Rutherford status distribution at 6 weeks after the surgery and the occurrence of a reintervention.

Utility values were assigned to each health state of the HSTM to calculate QALYs. All (dis)utility values were obtained from literature. For the R0-3 and R4-6 health states, utility values were calculated as the weighted average of the proportion of individual having each Rutherford status at 6 weeks by the utility value assigned to each Rutherford status [19–21].

The effect of reintervention and major amputation on quality of life was incorporated as disutility values applied on the R0-3 and R4-6 health state utility values [22]. We assumed that individuals had a lower quality of life after a reintervention (or an amputation) compared to when they did not experience any reintervention (or amputation), and applied the post-event quality of life loss from van Stel et al. [22] on the R0-3 and R4-6 health state utility values to obtain the post-reintervention (post-amputation) utility values.

### Resource use and costs

Health care costs related to the procedures, hospitalisation, monitoring, medication, reinterventions, and (post-)amputation were considered. Costs were expressed in Euros and indexed to their 2022 value.

The costs of procedures contained the costs of using the operating room and material costs (S.M.A.R.T.^®^ bare metal stents, Advanta v12 covered stents or surgical graft). For CERAB and KS, procedure and stents costs were estimated based on the CERAB and KS datasets. The procedural time was assumed equal for CERAB and KS. OSR procedural time was obtained from literature [23]. The prices and types of stents and graft used for the procedures were obtained from the hospital gathering evidence on CERAB.

For the CERAB and KS strategies, length of stay at the regular ward and at the ICU, and the probability of ICU admission were estimated using the CERAB dataset. Length of stay at the hospital and ICU for the OSR strategy were based on literature [2, 24].

Individuals in the CERAB and KS strategies were assumed to be monitored through duplex ultrasound at 6 weeks, 6 months, and every year thereafter. Individuals in the OSR strategy were only monitored through duplex ultrasound at one and five year after the procedure. The costs of the duplex ultrasound was equal to the costs of an outpatient clinic visit and an echography obtained from the Dutch guidelines [10].

Individuals in all strategies were assumed to be treated lifelong with statins. Individuals in the CERAB and KS strategies were assumed to receive three months dual antiplatelet (clopidogrel and carbasalate calcium) followed by single antiplatelet therapy (clopidogrel). Individuals in the OSR strategy were assumed to receive single antiplatelet therapy.

The costs of reintervention for CERAB and KS were calculated as the weighted average of the costs of the different types of reintervention occurring in the CERAB dataset (thrombolysis, surgical embolectomy, percutaneous transluminal angioplasty, bypass, additional stenting, or a combination of those). The costs of reintervention in the OSR strategy were based on a weighted average of the costs of a selection of reinterventions (incisional hernia, wound infection, thrombolysis, and ileus) described in a study on OSR for abdominal aortic aneurysm [25] and clinical expert opinion (MR). The costs of reintervention and amputation in all strategies were calculated based unit prices obtained from the site which collected data on CERAB. The post-amputation costs were obtained from literature [26].

### Model assumptions

The following assumptions were made to design the HE model based on clinical expert (MR) opinion:

1. The Rutherford distribution 6 weeks after the surgical intervention was assumed to be the same in all three strategies,
2. Individuals could only experience one reintervention because we observed that most individuals in the CERAB dataset (> 90%) either did not experience a reintervention or experienced only one reintervention,
3. The probability of requiring a reintervention was independent of Rutherford status. We made this assumption because the number of reinterventions observed per Rutherford status was too limited to estimate stable model parameters,
4. The probability of requiring a reintervention in the OSR strategy was lower than the probability of experiencing a reintervention in the endovascular strategies,
5. Only individuals with a Rutherford status of 4–6 were at risk of requiring an amputation,
6. OSR surgery had a technical success rate of 100%, CERAB and KS technical success rates were calculated based on CERAB individual-level data.

### Analyses

A cohort of 1,000 individuals was simulated. Total health effects and costs were calculated for each strategy. Incremental QALYs and costs of OSR and CERAB versus KS were calculated and fully incremental cost-effectiveness ratios (ICER) were calculated. An ICER is calculated as the difference in costs divided by the difference in QALYs between two strategies. All results in this manuscript were obtained from probabilistic analyses (PA) using Monte Carlo simulation containing 5,000 iterations [27] (see Appendix for detailed methods). All results are presented as mean (95% confidence interval). Probabilistic results were plotted in an incremental cost-effectiveness plane. Cost-effectiveness acceptability curves were plotted to display the probability that each strategy was cost-effective at different willingness-to-pay thresholds. The willingness-to-pay thresholds represent the societal value of a QALY in Euros. A strategy was considered cost effective when its ICER was lower than a willingness-to-pay threshold of €50,000 per QALY, as recommended by the Dutch guidelines for diseases with a disease burden between 0.41 and 0.70 [28].

Due to the uncertainty surrounding the length of stay at the ICU after an OSR, we performed a sensitivity analysis assuming an ICU stay of two days after an OSR (base-case value was 5.3 (95% confidence interval: 4.0-6.8) [24]. Probabilistic one-way sensitivity analyses (POWA) using the 95% confidence interval of each parameter were performed to assess the impact of varying each model input on the results. In addition the cost-effectiveness of CERAB, KS, and OSR was assessed in the following subgroups: (1) individuals being 65 years old or older, (2) individuals younger than 65 years old, (3) individuals who already underwent a previous intervention in the target lesion, and (4) individuals who did not undergo a previous intervention in the target lesion. In all subgroup analyses, we assumed the same relative effectiveness of OSR versus CERAB as in the base case analysis because it was not possible to re-estimate it in all subgroups due to their size and non-overlapping population characteristics. Three scenario analyses in which the time horizon was extended to 10, 15 years, and lifetime were performed.

All analyses were performed using R version 4.3.2 [29]. All model inputs are provided in the Appendix. This HE evaluation is reported according to the CHEERS 2022 statement [30] and validation efforts are reported using the TECH-VER [31] and AdViSHE checklist [32] (Online Supplemental material).

## Results

### Base-case results

**Fig. 2:**
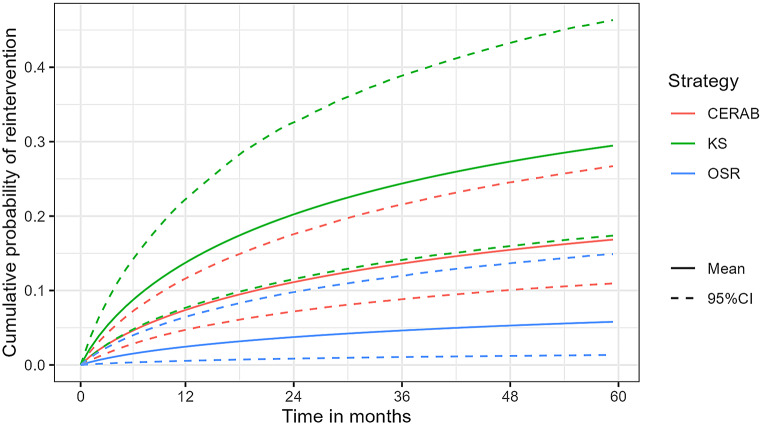
Cumulative probability of a reintervention per strategy. Abbreviations: CERAB = Covered Endovascular Reconstruction of the Aorto-iliac Bifurcation; CI = Confidence interval; KS = Kissing Stents; OSR = Open Surgical Repair

OSR resulted in the lowest probability of reintervention (6% (1 − 15%) and total LYs 4.23 (4.19–4.26) compared with CERAB (probability reintervention: 17% (11 − 27%); total LYs: 4.32 (4.24–4.35)) and KS (probability reintervention: 29% (17 − 46%); total LYs: 4.32 (4.24–4.35)) (Table 1; Fig. 2). Mean total QALYs per individual of OSR, KS, and CERAB were 2.89 (2.63–3.13), 2.88 (2.60–3.13), 2.92 (2.65–3.17).

**Table 1:** Basecase results

Strategy	Mean LY (95%CI)	Probability reintervention (95%CI)	Mean QALY (95%CI)	Mean costs (95%CI)	Incremental QALY	Incremental costs	ICER
KS	4.32 (4.24–4.35)	29% (17 − 46%)	2.88 (2.60–3.13)	€ 9,240 (€ 6,614 - € 13,176)	-	-	Start comparison
OSR	4.23 (4.19–4.26)	6% (1 − 15%)	2.89 (2.63–3.13)	€ 26,030 (€ 20,639 - € 32,000)	0.02	€ 16,790	Dominated
CERAB	4.32 (4.24–4.35)	17% (11 − 27%)	2.92 (2.65–3.17)	€ 14,564 (€ 10,466 - € 20,776)	0.05	€ 5,324	€ 110,201

Strategies are ordered in increasing mean QALYs

Abbreviations: CERAB = Covered Endovascular Reconstruction of the Aorto-iliac Bifurcation; CI = Confidence interval; KS = Kissing Stents; OSR = Open Surgical Repair

**Fig. 3:**
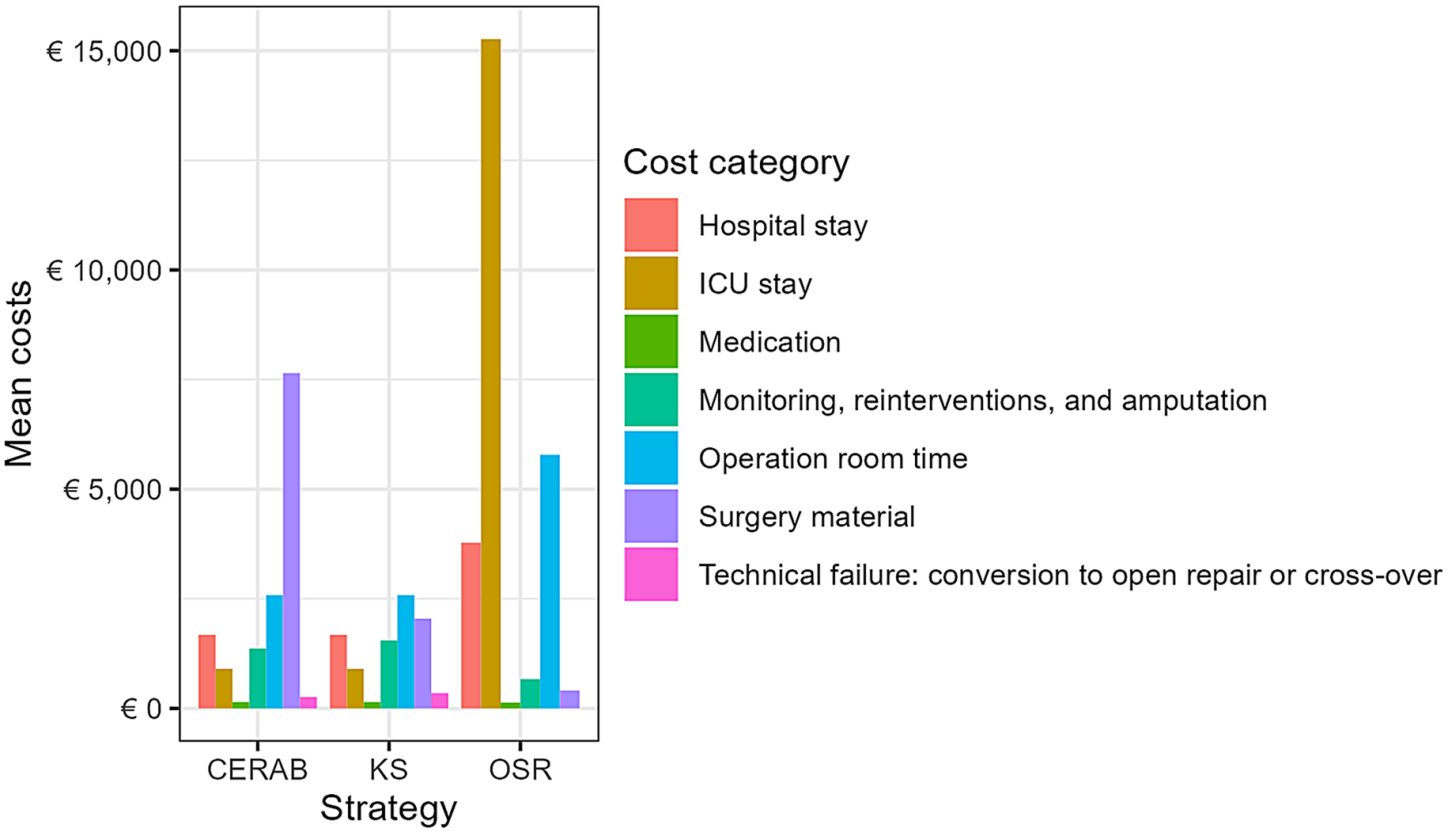
Disaggregated costs per strategy. Abbreviations: CERAB = Covered Endovascular Reconstruction of the Aorto-iliac Bifurcation; KS = Kissing Stents; OSR = Open Surgical Repair

Mean total costs of OSR, KS, and CERAB were respectively € 26,030 (€ 20,639 - € 32,000), € 9,240 (€ 6,614 - € 13,176), € 14,564 (€ 10,466 - € 20,776). OSR led to higher costs as a consequence of longer ICU stay compared with CERAB and KS (Table 1; Fig. 3).

**Fig. 4:**
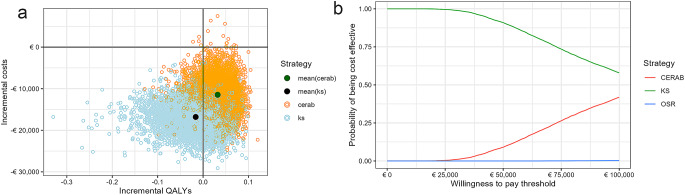
Probabilistic results. **A**: Incremental cost-effectiveness plane. **B**: Cost-effectiveness acceptability curves. Legend **A**: Incrementals were calculated versus Open Surgical Repair. Abbreviations: CERAB = Covered Endovascular Reconstruction of the Aorto-iliac Bifurcation; KS = Kissing Stents

OSR was dominated by CERAB since OSR resulted in lower QALYs gained and higher costs than CERAB. CERAB resulted in incremental QALYs and costs versus KS of respectively 0.048 (0.011–0.109) and € 5,324 (€ 2,938 - € 10,397). Hence, the ICER per QALY of CERAB versus KS was € 110,201, which was above the willingness-to-pay threshold of €50,000 per QALY. The probabilities of CERAB being cost effective at the willingness-to-pay thresholds of € 20,000, € 50,000, and € 80,000 per QALY were 0%, 9%, 30%. These probabilities were respectively 100%, 91%, 70% for KS (Fig. 4).

### Sensitivity and subgroup analyses

The POWA indicates that the number of stents used for the CERAB procedure was the most influential parameter on the results when comparing CERAB to KS. The number of days at the ICU after an OSR was the most influential parameter on the results when comparing OSR to KS. Assuming that the length of stay at the ICU was only two days does not change the conclusion of the HE evaluation (Appendix). The subgroup analyses show that CERAB may be cost effective versus KS in individuals younger than 65 years old as CERAB leads to higher health benefits compared with KS in this subgroup than in older individuals. The scenario analyses show that CERAB may be cost-effectiveness compared with KS and OSR over a longer time horizon if its benefits persists over time (Table 2).

**Table 2:** Subgroup and scenario analyses restsul

Subgroup or Scenario	Strategy	Mean LY (95%CI)	Probability reintervention (95%CI)	Mean QALY (95%CI)	Mean costs (95%CI)	Incremental QALY	Incremental costs	ICER
Basecase	KS	4.32 (4.24–4.35)	29% (17 − 46%)	2.88 (2.6–3.13)	€ 9,240 (€ 6,614 - € 13,176)	-	-	Start comparison
Basecase	OSR	4.23 (4.19–4.26)	6% (1 − 15%)	2.89 (2.63–3.13)	€ 26,030 (€ 20,639 - € 32,000)	0.02	€ 16,790	Dominated
Basecase	CERAB	4.32 (4.24–4.35)	17% (11 − 27%)	2.92 (2.65–3.17)	€ 14,564 (€ 10,466 - € 20,776)	0.05	€ 5,324	€ 110,201
65+	CERAB	3.77 (3.62–3.85)	8% (0 − 25%)	2.34 (1.85–2.74)	€ 17,149 (€ 11,497 - € 25,043)	-	-	Dominated
65+	KS	3.77 (3.62–3.85)	7% (0 − 25%)	2.35 (1.85–2.74)	€ 11,130 (€ 7,035 - € 17,535)	-	-	Start comparison
65+	OSR	3.72 (3.67–3.78)	10% (0 − 23%)	2.36 (1.91–2.71)	€ 27,610 (€ 21,199 - € 34,760)	0.01	€ 16,480	€ 1,577,465
65-	KS	4.66 (4.64–4.68)	34% (18 − 55%)	2.32 (1.52–3.02)	€ 7,876 (€ 5,915 - € 10,541)	-	-	Start comparison
65-	CERAB	4.66 (4.64–4.68)	21% (13 − 35%)	2.51 (1.73–3.15)	€ 12,856 (€ 9,633 - € 17,759)	0.19	€ 4,980	€ 25,794
65-	OSR	4.54 (4.49–4.57)	7% (2 − 20%)	2.63 (1.83–3.24)	€ 26,612 (€ 21,086 - € 32,828)	0.12	€ 13,756	€ 119,202
Previous intervention	KS	3.77 (3.62–3.85)	46% (26 − 69%)	2.36 (2.03–2.68)	€ 11,125 (€ 7,260 - € 17,399)	-	-	Start comparison
Previous intervention	CERAB	3.77 (3.62–3.85)	31% (18 − 51%)	2.43 (2.11–2.73)	€ 16,861 (€ 11,311 - € 24,746)	0.06	€ 5,736	€ 88,682
Previous intervention	OSR	3.72 (3.67–3.78)	12% (3 − 30%)	2.45 (2.13–2.74)	€ 26,308 (€ 20,760 - € 32,292)	0.03	€ 9,447	€ 361,791
No previous intervention	OSR	3.72 (3.67–3.78)	3% (1 − 11%)	2.48 (2.15–2.76)	€ 25,867 (€ 20,365 - € 31,814)	-	-	Dominated
No previous intervention	KS	3.77 (3.62–3.85)	15% (5 − 35%)	2.48 (2.15–2.77)	€ 10,633 (€ 6,795 - € 16,955)	-	-	Start comparison
No previous intervention	CERAB	3.77 (3.62–3.85)	10% (5 − 23%)	2.5 (2.17–2.79)	€ 16,556 (€ 11,083 - € 24,360)	0.02	€ 5,922	€ 394,117
Time horizon 10 years	KS	7.41 (7.27–7.49)	36% (21 − 55%)	4.87 (4.35–5.33)	€ 10,035 (€ 7,402 - € 13,973)	-	-	Start comparison
Time horizon 10 years	OSR	7.26 (7.18–7.33)	7% (2 − 19%)	4.93 (4.43–5.36)	€ 26,236 (€ 20,823 - € 32,186)	0.06	€ 16,202	Dominated
Time horizon 10 years	CERAB	7.41 (7.27–7.49)	21% (14 − 34%)	4.96 (4.46–5.4)	€ 15,354 (€ 11,266 - € 21,577)	0.09	€ 5,320	€ 57,015
Time horizon 15 years	KS	9.56 (9.38–9.68)	38% (23 − 59%)	6.24 (5.52–6.86)	€ 10,553 (€ 7,888 - € 14,504)	-	-	Start comparison
Time horizon 15 years	OSR	9.36 (9.24–9.47)	8% (2 − 21%)	6.33 (5.55–6.92)	€ 26,420 (€ 20,967 - € 32,416)	0.09	€ 15,866	Dominated
Time horizon 15 years	CERAB	9.56 (9.38–9.68)	23% (14 − 37%)	6.36 (5.63–6.96)	€ 15,892 (€ 11,794 - € 22,137)	0.12	€ 5,339	€ 43,453
Lifetime time horizon	KS	12.09 (11.84–12.29)	41% (24 − 61%)	7.85 (6.83–8.67)	€ 11,132 (€ 8,390 - € 15,123)	-	-	Start comparison
Lifetime time horizon	OSR	11.84 (11.65–12.03)	9% (2 − 23%)	7.96 (6.65–8.78)	€ 26,708 (€ 21,114 - € 32,821)	0.12	€ 15,576	Dominated
Lifetime time horizon	CERAB	12.09 (11.84–12.29)	25% (15 − 40%)	8 (6.88–8.81)	€ 16,522 (€ 12,325 - € 22,835)	0.15	€ 5,390	€ 34,992

In each subgroup ans scenario analyses, strategies are ordered in increasing mean QALYs Abbreviations: CERAB = Covered Endovascular Reconstruction of the Aorto-iliac Bifurcation; CI = Confidence interval; KS = Kissing Stents; OSR = Open Surgical Repair

## Discussion

This HE evaluation assesses the cost-effectiveness of CERAB versus KS and OSR for the treatment of extensive AIOD. Even though OSR led to the lowest number of reintervention per person, OSR was dominated - more costly and less effective - by CERAB. This was caused by the higher probability of death and higher hospital costs of OSR compared to CERAB. CERAB provided limited additional health benefits (0.05 QALYs) compared to KS, due to a reduction in the number of reinterventions but increased health care costs (€ 5,324), resulting in ICER of € 110,201 for CERAB compared to KS. Consequently, CERAB is unlikely to be cost effective compared to KS when considering a willingness-to-pay threshold of € 50,000 per QALY.

Our results should be interpreted in light of the following limitations. Firstly, there is no direct comparison of CERAB versus KS nor OSR, and the available evidence only allowed to adjust the comparison for a limited number of variables. Hence, we cannot exclude that the current comparisons are biased due to unmeasured confounding. Secondly, the model structure allowed individuals to experience a single reintervention, while in practice individuals are at risk of experiencing subsequent reinterventions. Evidence concerning the risk of experiencing multiple reinterventions is however lacking. This assumption was considered conservative since the prevention (or postponement) of a first reintervention prevents (or postpones) the occurrence of subsequent reinterventions. Thirdly, the health-state-related quality of life was determined based on the distribution of Rutherford status 6-weeks after the CERAB surgery while this distribution may vary over time. Fourthly, the evidence concerning CERAB was obtained from a single pioneering centre in this type of surgery, which may have negatively influenced the early results of CERAB due to the learning curve associated with using a new procedure. Finally, we made the assumption that simulated individuals received three months dual antiplatelet followed by single antiplatelet therapy in the CERAB and KS strategies, but the most recent European guideline for the treatment of peripheral arterial disease and intermittent claudication recommends prescribing low dose rivaroxaban and aspirin after revascularisation [33]. This may have led to an underestimation of the total costs of the CERAB and KS strategies. This is however unlikely to affect the conclusions of our analyses when considering that OSR is €16,790 and €11,466 more expensive than KS and CERAB respectively, and that CERAB is not cost effective versus KS using Dutch willingness-to-pay thresholds.

To the best of our knowledge, this is the first health economic evaluation of CERAB versus KS and OSR for treating extensive AIOD. Previous studies have mainly focused on evaluating the clinical effectiveness of CERAB, KS, and OSR through observational studies [1, 2, 13]. Few of these studies have compared the clinical outcomes of endovascular procedures and OSR using observational research design [14, 24, 34–38], but none have estimated the relative effectiveness of CERAB versus KS or OSR. Beside the absence of relative effectiveness of CERAB versus KS and OSR, evidence on the economic impact of each of these treatments is scarce. A previous retrospective study investigated the costs of OSR and endovascular treatment and showed that the intervention costs of OSR were almost twice as high as the costs of endovascular treatment [34], due to longer hospital and intensive care unit stay associated with OSR compared to endovascular procedures. Even though our findings are in line with this previous study and may be relevant to countries with a similar healthcare system as the Netherlands, they may not directly be generalised to countries with different healthcare systems.

The estimated limited health benefits provided by CERAB versus KS (0.05 QALYs) drive the ICER above the Dutch willingness-to-pay threshold, even though the absolute increase in costs due to the use of additional stents to perform CERAB versus KS is limited (€ 5,324). Further benefits for the hospital, such as less operation and personnel time due to a reduced number of reinterventions versus KS (of 12%), are however not captured in the current comparison. Consequently, this ICER may underestimate the value of CERAB versus KS.

## Conclusion

This health economic evaluation shows that CERAB leads to slightly higher health benefits (QALYs) and lower health care costs compared with OSR for treating extensive AIOD. Compared to KS, CERAB provides limited health benefits at slightly increased costs, and is unlikely to be cost effective. These results should be interpreted with caution since the relative effectiveness of CERAB versus KS and OSR was estimated based on indirect comparisons. Once new evidence from the EVOCC or other trials becomes available, our analyses can be easily updated to provide more accurate outcomes.

## Electronic supplementary material

Below is the link to the electronic supplementary material.

Supplementary Material 1

## Data Availability

The data and health economic model are not openly available and may be provided upon request.

## Declarations

### Artificial intelligence generated content

The authors did not use any Artificial Intelligence for any aspects of this research.

## References

[1] Salem, M., Hosny, M.S., Francia, F., Sallam, M., Saratzis, A., Saha, P., et al.: Management of extensive aorto-iliac disease: A systematic review and meta-analysis of 9319 patients. Cardiovasc. Interv. Radiol. **44**, 1518–1535 (2021) 10.1007/s00270-021-02785-6 34279686

[2] Premaratne, S., Newman, J., Hobbs, S., Garnham, A., Wall, M.: Meta-analysis of direct surgical versus endovascular revascularization for aortoiliac occlusive disease. J. Vasc. Surg. **72**(2), 726–737 (2020) 32171442 10.1016/j.jvs.2019.12.035

[3] Aboyans, V., Ricco, J., MLEL Bartelink, M., Björck, M., Brodmann, M., Cohnert, T., et al.: Editor’s choice 2017 ESC guidelines on the diagnosis and treatment of peripheral arterial diseases, in collaboration with the European society for vascular surgery (ESVS). Eur. J. Vascular Endovascular Surgery: Official J. Eur. Soc. Vascular Surg. **55** 3 305–368 10.1016/j.ejvs.2017.07.018 (2018) 10.1016/j.ejvs.2017.07.018 28851596

[4] Reijnen, M.M.: Update on covered endovascular reconstruction of the aortic bifurcation. Vascular. **28**(3), 225–232 (2020) 31896301 10.1177/1708538119896197

[5] Goverde, P., Grimme, F., Verbruggen, P., Reijnen, M.: Covered endovascular reconstruction of aortic bifurcation (CERAB) technique: A new approach in treating extensive aortoiliac occlusive disease. J. Cardiovasc. Surg. (Torino). **54**(3), 383–387 (2013) 23640357

[6] Raad voor het Regeringsbeleid] [Wetenschappelijke. Kiezen Voor Houdbare Zorg. Mensen, Middelen En Maatschappelijk Draagvlak, Wrr-Rapport 104. WRR: (2022). https://www.wrr.nl/adviesprojecten/houdbare-zorg/documenten/rapporten/2021/09/15/kiezen-voor-houdbare-zorg

[7] Indes, J.E., Mandawat, A., Tuggle, C.T., Muhs, B., Sosa, J.A.: Endovascular procedures for aorto-iliac occlusive disease are associated with superior short-term clinical and economic outcomes compared with open surgery in the inpatient population. J. Vasc. Surg. **52**(5), 1173–1179 (2010) 20691560 10.1016/j.jvs.2010.05.100

[8] Indes, J.E., Tuggle, C.T., Mandawat, A., Sosa, J.A.: Age-stratified outcomes in elderly patients undergoing open and endovascular procedures for aortoiliac occlusive disease. Surgery. **148**(2), 420–428 (2010) 20580044 10.1016/j.surg.2010.05.008

[9] Drummond, M.F., Sculpher, M.J., Claxton, K., Stoddart, G.L., Torrance, G.W.: Methods for the Economic Evaluation of Health Care Programmes. Oxford University Press (2015)

[10] Dutch Health Care institute: Guidelines for Performing Health Economic Evaluation. Dutch Health Care institute (2016)

[11] Rouwenhorst, K.B., Abdelbaqy, O.M., Van der Veen, D., van Rijswijk, R.E., Holewijn, S., Reijnen, M.M.: Long-term outcomes of the covered endovascular reconstruction of the aortic bifurcation (CERAB) technique in patients with aorto-iliac occlusive disease. J. Endovascular Therapy Published Online. 15266028231166539 (2023). 10.1177/15266028231166539 10.1177/15266028231166539 37114939

[12] Taeymans, K., Groot Jebbink, E., Holewijn, S., Martens, J.M., Versluis, M., Goverde, P.C., et al.: Three-year outcome of the covered endovascular reconstruction of the aortic bifurcation technique for aortoiliac occlusive disease. J. Vasc. Surg. **67**(5), 1438–1447 (2018) 29169878 10.1016/j.jvs.2017.09.015

[13] Groot Jebbink, E., Holewijn, S., Versluis, M., Grimme, F., Hinnen, J.W., Sixt, S., et al.: Meta-analysis of individual patient data after kissing stent treatment for aortoiliac occlusive disease. J. Endovasc. Ther. **26**(1), 31–40 (2019) 30499352 10.1177/1526602818810535 PMC6330696

[14] Dorigo, W., Piffaretti, G., Benedetto, F., Tarallo, A., Castelli, P., Spinelli, F., et al.: A comparison between aortobifemoral bypass and aortoiliac kissing stents in patients with complex aortoiliac obstructive disease. J. Vasc. Surg. **65**(1), 99–107 (2017) 27633164 10.1016/j.jvs.2016.06.107

[15] Sekhon, J.S.: Multivariate and Propensity Score Matching Software with Automated Balance Optimization: The Matching Package for R. J. Stat. Softw. **42**(1), 1–52 (2011). 10.18637/jss.v042.i07

[16] Phillippo, D., Ades, T., Dias, S., Palmer, S., Abrams, K.R., Welton, N.: NICE DSU technical support document 18: Methods for population-adjusted indirect comparisons in submissions to NICE. Published online 2016.

[17] Saratzis, A., Salem, M., Sabbagh, C., Abisi, S., Huasen, B., Egun, A., et al.: Treatment of aortoiliac occlusive disease with the covered endovascular reconstruction of the aortic bifurcation (CERAB) technique: Results of a UK multicenter study. J. Endovasc. Ther. **28**(5), 737–745 (2021) 34160321 10.1177/15266028211025028

[18] Centraal Bureau voor Statistiek: (2023). https://www.cbs.nl/en-gb/. Accessed March 28, 2023

[19] Chalmers, N., Walker, P.T., Belli, A.M., Thorpe, A.P., Sidhu, P.S., Robinson, G., et al.: Randomized trial of the SMART stent versus balloon angioplasty in long superficial femoral artery lesions: The SUPER study. Cardiovasc. Interv. Radiol. **36**(2), 353–361 (2013) 10.1007/s00270-012-0492-z 23070104

[20] de Vries, M., Ouwendijk, R., Kessels, A.G., de Haan, M.W., Flobbe, K., Hunink, M.G., et al.: Comparison of generic and disease-specific questionnaires for the assessment of quality of life in patients with peripheral arterial disease. J. Vasc. Surg. **41**(2), 261–268 (2005) 15768008 10.1016/j.jvs.2004.11.022

[21] Versteegh, M.M., Vermeulen, K.M., Evers, S.M., De Wit, G.A., Prenger, R., Stolk, E.A.: Dutch tariff for the five-level version of EQ-5D. Value Health. **19**(4), 343–352 (2016) 27325326 10.1016/j.jval.2016.01.003

[22] Van Stel, H.F., Busschbach, J.J., Hunink, M.M., Buskens, E.: Impact of secondary cardiovascular events on health status. Value Health. **15**(1), 175–182 (2012) 22264986 10.1016/j.jval.2011.09.004

[23] Tong, Y., Khachane, A., Ibrahim, M., Jacob, T., Shiferson, A., Almadani, M., et al.: Open abdominal aortic repair in the current era has more complications for occlusive disease than for aneurysm repair. J. Vasc. Surg. **77**(2), 432–439 (2023) 36130697 10.1016/j.jvs.2022.09.010

[24] Mayor, J., Branco, B.C., Chung, J., Montero-Baker, M.F., Kougias, P., Mills Sr, J.L., et al.: Outcome comparison between open and endovascular management of TASC II d aortoiliac occlusive disease. Ann. Vasc. Surg. **61**, 65–71 (2019) 31394230 10.1016/j.avsg.2019.06.005

[25] van Schaik, T.G., Yeung, K.K., Verhagen, H.J., de Bruin, J.L., van Sambeek, M.R., Balm, R., et al.: Long-term survival and secondary procedures after open or endovascular repair of abdominal aortic aneurysms. J. Vasc. Surg. **66**(5), 1379–1389 (2017) 29061270 10.1016/j.jvs.2017.05.122

[26] Oostenbrink, J.B., Tangelder, M.J., Busschbach, J.J., van Hout, B.A., Buskens, E., Algra, A., et al.: Cost-effectiveness of oral anticoagulants versus aspirin in patients after infrainguinal bypass grafting surgery. J. Vasc. Surg. **34**(2), 254–262 (2001) 11496277 10.1067/mva.2001.115961

[27] Thompson, K.M., Graham, J.D.: Going beyond the single number: Using probabilistic risk assessment to improve risk management. Hum. Ecol. Risk Assessment: Int. J. **2**(4), 1008–1034 (1996)

[28] Zwaap, J., Knies, S., van der Meijden, C., Staal, P.: Heiden Luuk Van Der. Rapport Kosteneffectiviteit in De Praktijk. Dutch Health Care institute (2015)

[29] R Core Team. R: A Language and Environment for Statistical Computing. R Foundation for Statistical Computing: (2022). https://www.R-project.org/

[30] Husereau, D., Drummond, M., Augustovski, F., de Bekker-Grob, E., Briggs, A.H., Carswell, C., et al.: Consolidated health economic evaluation reporting standards 2022 (CHEERS 2022) statement: Updated reporting guidance for health economic evaluations. Int. J. Technol. Assess. Health Care. **38**(1), e13 (2022) 35007499 10.1017/S0266462321001732

[31] Büyükkaramikli, N.C., Rutten-van Mölken, M.P., Severens, J.L., Al, M.: TECH-VER: A verification checklist to reduce errors in models and improve their credibility. Pharmacoeconomics. **37**, 1391–1408 (2019) 31705406 10.1007/s40273-019-00844-y PMC6860463

[32] Vemer, P., Corro Ramos, I., Van Voorn, G., Al, M., Feenstra, T., AdViSHE: A validation-assessment tool of health-economic models for decision makers and model users. Pharmacoeconomics. **34**, 349–361 (2016) 26660529 10.1007/s40273-015-0327-2 PMC4796331

[33] Nordanstig, J., Behrendt, C. A., Baumgartner, I., Belch, J., Bäck, M., Fitridge, R., Hinchliffe, R., Lejay, A., Mills, J. L., Rother, U., Sigvant, B., Spanos, K., Szeberin Z., van de Water W.; ESVS Guidelines Committee; Antoniou GA, Björck M, Gonçalves FB, Coscas R, Dias NV: Van Herzeele I, Lepidi S, Mees BME, Resch TA, Ricco JB, Trimarchi S, Twine CP, Tulamo R, Wanhainen A; Document Reviewers; Boyle JR, Brodmann M, Dardik A, Dick F, Goëffic Y, Holden A, Kakkos SK, Kolh P, McDermott MM. Editor’s Choice -- European Society for Vascular Surgery (ESVS) 2024 Clinical Practice Guidelines on the Management of Asymptomatic Lower Limb Peripheral Arterial Disease and Intermittent Claudication. Eur J Vasc Endovasc Surg.;67(1):9–96. (2024) 10.1016/j.ejvs.2023.08.067 37949800

[34] Rocha-Neves, J., Ferreira, A., Sousa, J., Pereira-Neves, A., Vidoedo, J., Alves, H., et al.: Endovascular approach versus aortobifemoral bypass grafting: Outcomes in extensive aortoiliac occlusive disease. Vasc. Endovascular. Surg. **54**(2), 102–110 (2020) 31746273 10.1177/1538574419888815

[35] Squizzato, F., D’Oria, M., Bozza, R., Porcellato, L., Grego, F., Lepidi, S.: Propensity-matched comparison of endovascular versus open reconstruction for TASC-II C/d aortoiliac occlusive disease. A ten-year single-center experience with self-expanding covered stents. Ann. Vasc. Surg. **71**, 84–95 (2021) 32927036 10.1016/j.avsg.2020.08.139

[36] Lee, C.W., Huh, U., Bae, M., Han, C., Kwon, H., Kim, G.: Comparison between kissing stents and direct surgical bypass for aortoiliac occlusive disease. J. Chest Surg. **56**(4), 264 (2023) 37096251 10.5090/jcs.23.012 PMC10345658

[37] Burke, C.R., Henke, P.K., Hernandez, R., Rectenwald, J.E., Krishnamurthy, V., Englesbe, M.J., et al.: A contemporary comparison of aortofemoral bypass and aortoiliac stenting in the treatment of aortoiliac occlusive disease. Ann. Vasc. Surg. **24**(1), 4–13 (2010) 20122461 10.1016/j.avsg.2009.09.005

[38] Gabel, J.A., Kiang, S.C., Abou-Zamzam, A.M. Jr., Oyoyo, U.E., Teruya, T.H., Tomihama, R.T.: Trans-atlantic inter-society consensus class d aortoiliac lesions: A comparison of endovascular and open surgical outcomes. Am. J. Roentgenol. **213**(3), 696–701 (2019) 31120778 10.2214/AJR.18.20918

