# Supplementary material for: Cost-effectiveness analysis of the covered endovascular reconstruction of the aortic bifurcation versus kissing stents and open surgical repair for the treatment of aorto-iliac occlusive disease
Source: Eur J Health Econ. 2025 Jul 30;27(2):277–87. doi: 10.1007/s10198-025-01792-5 (PMC13046675; doi:10.1007/s10198-025-01792-5)
Supplement: Supplementary file 1 — Supplementary Material 1 [file 10198_2025_1792_MOESM1_ESM.docx]

Cost-effectiveness analysis of the Covered Endovascular Reconstruction of the Aortic Bifurcation versus Kissing Stents and Open Surgical Repair for the treatment of Aorto-iliac Occlusive Disease - Appendix

X.G.L.V. Pouwels, S. Holewijn, D. van der Veen, M. Gonzalez-Urquijo, M.M.P.J. Reijnen, H. Koffijberg

2024-02-13

Table of Contents

[Detailed methods section 3](#_Toc158706636)

[Multiple imputation 3](#_Toc158706637)

[Probability of target lesion reintervention: CERAB versus KS 5](#_Toc158706638)

[Probability of target lesion reintervention: Open repair versus CERAB 34](#_Toc158706639)

[Other transition probabilities 36](#_Toc158706640)

[Probability of amputation 36](#_Toc158706641)

[Probability of disease-related death 37](#_Toc158706642)

[Probability of death due to other causes 37](#_Toc158706643)

[Utility values 38](#_Toc158706644)

[Resource use and costs 39](#_Toc158706645)

[Model inputs overview 40](#_Toc158706646)

[Probabilistic analysis 52](#_Toc158706647)

[Probabilistic one-way sensitivity analyses 52](#_Toc158706648)

[Subgroup analyses 52](#_Toc158706649)

[Health economic model validation 53](#_Toc158706650)

[Part A: Validation of the conceptual model 53](#_Toc158706651)

[Part B: Input data validation 53](#_Toc158706652)

[Part C: Validation of the computerized model 54](#_Toc158706653)

[Part D: Operational validation 55](#_Toc158706654)

[Part E: Other validation techniques 56](#_Toc158706655)

[Determining the willingness to pay threshold 57](#_Toc158706656)

[Additional results 58](#_Toc158706657)

[Cost effectiveness plane 58](#_Toc158706658)

[Sensivity analysis shorter length of stay at the ICU 59](#_Toc158706659)

[CERAB versus KS: probabilistic one-way sensitivity analysis 60](#_Toc158706660)

[OSR versus KS: probabilistic one-way sensitivity analysis 61](#_Toc158706661)

[Subgroup analyses: probability of reintervention 62](#_Toc158706662)

[Convergence incremental costs & QALYs 66](#_Toc158706663)

[R session information 67](#_Toc158706664)

[References 69](#_Toc158706665)

# Detailed methods section

## Multiple imputation

Missing data were multiply imputed using the mice R package^1^. The following missing data were imputed in the following order CERAB dataset: age when undergoing the procedure, the TASC classification, Rutherford status pre-operation, whether the individual had already undergone another endovascular procedure, the CERAB procedure time, the number of stents used during the CERAB, the number of admission days at the hospital, and the Rutherford status six weeks after the procedure. Additionally, missing Rutherford statuses during follow-up, which were used to calculate the probability of progression and regression in rutherford status over time, were imputed using the last observation carried forward (we also multiply imputed these values using the jomo R package^2^, which led to the same results).
In the Kissing Stent (KS) dataset, the following data were imputed in the following order: age when undergoing the procedure, the TASC classification, Rutherford status pre-operation, whether the individual had already undergone another endovascular procedure, whether the individual underwent a reintervention during follow-up (otherwise the individual was censored), and the time until reintervention or censoring.
Since different variables were imputed in each dataset, multiple imputation was performed per dataset. Variables’ values were imputed using information from all previously (complete or) imputed variables. The tables below provide an overview of the proportion of missing data per variable in each dataset.

Overview of missing data in the CERAB dataset (total N = 158)

|  | N missing | Percentage missing |
| --- | --- | --- |
| age procedure | 0 | 0 % |
| TASC class | 0 | 0 % |
| Rutherford pre-procedure | 0 | 0 % |
| former endovasc | 1 | 1 % |
| procedure time | 33 | 21 % |
| totalstents | 4 | 3 % |
| admission days | 4 | 3 % |
| Rutherford 6w | 31 | 20 % |

Overview of missing Rutherford data in the CERAB dataset in individuals with technical success only

|  | N missing | N total | Percentage missing |
| --- | --- | --- | --- |
| Rutherford_6mt | 44 | 150 | 29 % |
| Rutherford_12mt | 35 | 150 | 23 % |
| Rutherford_2yr | 22 | 150 | 15 % |
| Rutherford_3yr | 24 | 150 | 16 % |
| Rutherford_4yr | 23 | 150 | 15 % |
| Rutherford_5yr | 22 | 150 | 15 % |
| Rutherford_10yr | 12 | 150 | 8 % |

Overview of missing data in the KS dataset (total N = 605)

|  | N missing | Percentage missing |
| --- | --- | --- |
| age procedure | 1 | 0 % |
| TASC class | 5 | 1 % |
| Rutherford pre-procedure | 11 | 2 % |
| Previous intervention | 166 | 27 % |
| Reintervention (Yes/No) | 4 | 1 % |
| Time to reintervention / censoring | 17 | 3 % |

*Abbreviation: TASC = TransAtlantic interSociety Consensus*

## Probability of target lesion reintervention: CERAB versus KS

We were not able to use information from all individuals included in the KS dataset due to different definitions of patency and reinterventions in the CERAB and KS datasets (and among studies included in the KS dataset^3^). We selected individuals who were included in Hinnen et al.^4^ and Dorigo et al.^5^ since primary patency loss was defined as the occurrence of reintervention in the target lesion in these studies. To estimate the (relative) effectiveness of CERAB versus KS, we compared the occurrence of target lesion reintervention in CERAB to the occurrence of reintervention in the combined Hinnen et al.^4^ and Dorigo et al.^5^ studies. We also attempted to compare the occurrence of a target lesion reintervention with the other studies included in the KS dataset but this resulted in less balanced datasets concerning the baseline characteristics of the CERAB group and the matched KS group (results not shown) . The procedure described here below was performed in all ten imputed datasets.
To adjust for differences in baseline characteristics between the CERAB and KS groups, we first matched the KS group to the CERAB group (1:1 with replacement) using a genetic matching (GenMatch) algorithm (propensity score matching was also applied but led to less balanced groups in general, hence the results are not provided here; see the table below to see the difference in Kolmorogov-Smirnov bootstrapped p-value between no matching and matching using propensity score matching and Genetic Matching). Baseline characteristics used in the matching algorithm were selected based on clinical expert opinion (MR). The following baseline characteristics were included in the matching algorithm: age when undergoing the procedure, TASC classification, pre-procedure Rutherford status, and whether individuals underwent a previous intervention. A propensity score was calculated using these characteristics and both the propensity score and the individual baseline characteristics were included in the GenMatch algorithm. Balance of baseline characteristics between the CERAB group and the matched KS group was then assessed using visual inspection of frequency histograms (categorical variables) or QQ-plot (continuous variables) and the Kolmorogov-Smirnov bootstrapped p-value. The balance statistics before and after matching and QQ-plots for each baseline characteristics used in the GenMatch are described in the figures and tables below.

Baseline Kolmorogov-Smirnov p-value per matching variable before and after matching in all 10 imputed datasets

| Imputation | Variable | KS boot. p-value not matched | KS boot. p-value PS matched | KS boot. p-value GenMatched |
| --- | --- | --- | --- | --- |
| 1 | TASC | 0.000 | 0.958 | 0.960 |
| 1 | Age | 0.741 | 0.315 | 0.578 |
| 1 | Rutherford | 0.281 | 0.964 | 0.901 |
| 1 | Previous intervention | 0.037 | 1.000 | 0.853 |
| 2 | TASC | 0.000 | 0.861 | 0.964 |
| 2 | Age | 0.753 | 0.791 | 0.857 |
| 2 | Rutherford | 0.232 | 0.950 | 0.903 |
| 2 | Previous intervention | 0.088 | 1.000 | 1.000 |
| 3 | TASC | 0.000 | 0.956 | 0.998 |
| 3 | Age | 0.718 | 0.583 | 0.666 |
| 3 | Rutherford | 0.189 | 0.996 | 0.904 |
| 3 | Previous intervention | 0.048 | 1.000 | 1.000 |
| 4 | TASC | 0.000 | 0.883 | 0.999 |
| 4 | Age | 0.676 | 0.670 | 0.853 |
| 4 | Rutherford | 0.225 | 0.989 | 0.993 |
| 4 | Previous intervention | 0.042 | 1.000 | 0.941 |
| 5 | TASC | 0.000 | 0.730 | 0.965 |
| 5 | Age | 0.644 | 0.870 | 0.930 |
| 5 | Rutherford | 0.193 | 0.998 | 0.993 |
| 5 | Previous intervention | 0.074 | 1.000 | 1.000 |
| 6 | TASC | 0.000 | 0.880 | 0.954 |
| 6 | Age | 0.729 | 0.866 | 0.976 |
| 6 | Rutherford | 0.198 | 0.994 | 0.990 |
| 6 | Previous intervention | 0.445 | 1.000 | 0.839 |
| 7 | TASC | 0.000 | 0.857 | 0.970 |
| 7 | Age | 0.690 | 0.800 | 0.609 |
| 7 | Rutherford | 0.206 | 0.994 | 0.996 |
| 7 | Previous intervention | 0.313 | 1.000 | 1.000 |
| 8 | TASC | 0.000 | 0.869 | 0.951 |
| 8 | Age | 0.702 | 0.594 | 0.778 |
| 8 | Rutherford | 0.187 | 0.992 | 0.794 |
| 8 | Previous intervention | 0.109 | 1.000 | 1.000 |
| 9 | TASC | 0.000 | 0.721 | 0.853 |
| 9 | Age | 0.692 | 0.691 | 0.935 |
| 9 | Rutherford | 0.212 | 0.960 | 0.963 |
| 9 | Previous intervention | 0.316 | 1.000 | 1.000 |
| 10 | TASC | 0.000 | 0.886 | 0.999 |
| 10 | Age | 0.677 | 0.684 | 0.867 |
| 10 | Rutherford | 0.199 | 0.960 | 0.959 |
| 10 | Previous intervention | 0.044 | 1.000 | 0.821 |

*Abbreviations: GenMatched = Matched using a Genetic Matching algorithm; KS boot. p-value = Kolmorogov-Smirnov bootstrapped p-value; PS = propensity score; TASC = Trans-Atlantic Inter-Society Consensus classification*

**QQplots of age distribution among individuals who received the KS strategy versus the CERAB strategy before (right) and after (left) matching**


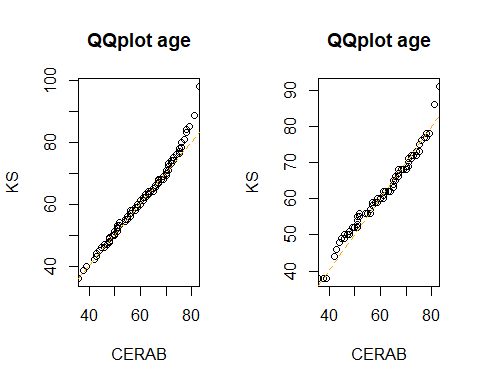

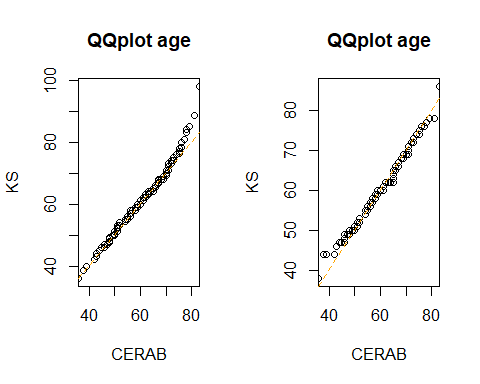

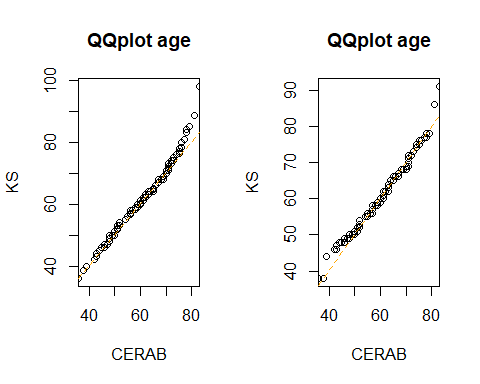

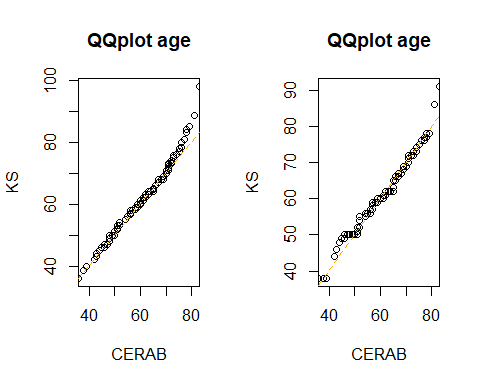

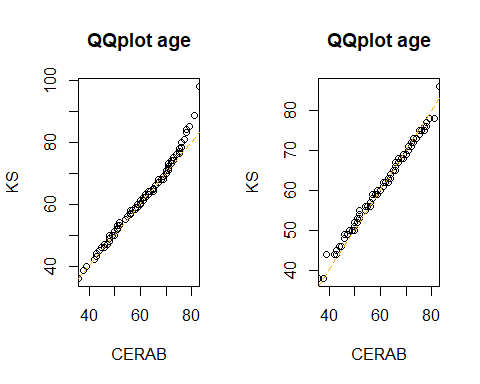

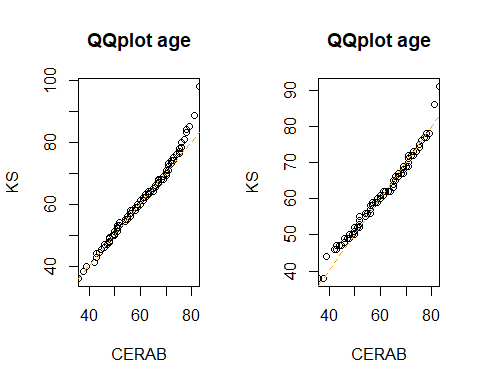

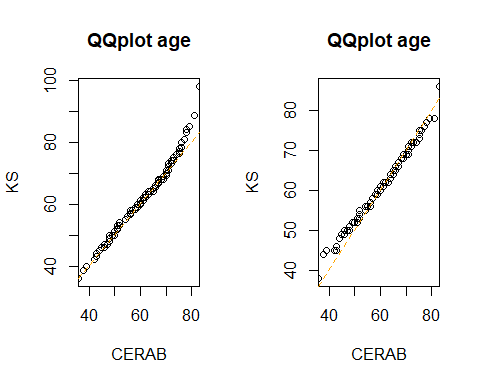

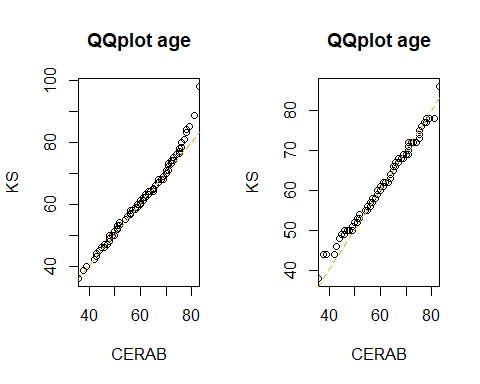

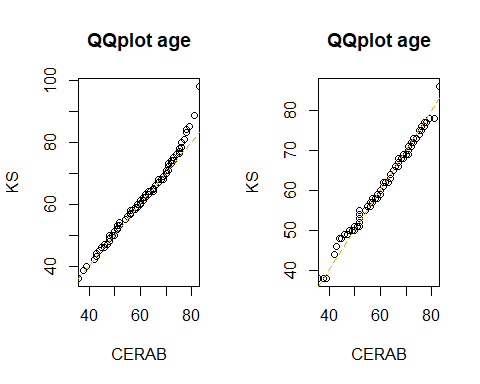

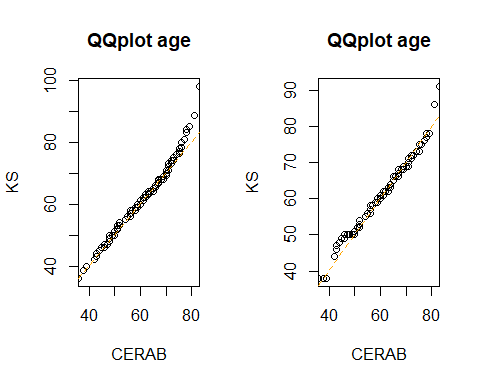


*Abbreviations: CERAB = Covered Endovascular Reconstruction of the Aorto-iliac Bifurcation; KS = Kissing Stents; QQ = quantile-quantile*

**TASC classification before (left) and after (right) matching in all ten imputed datasets**

## [[1]]


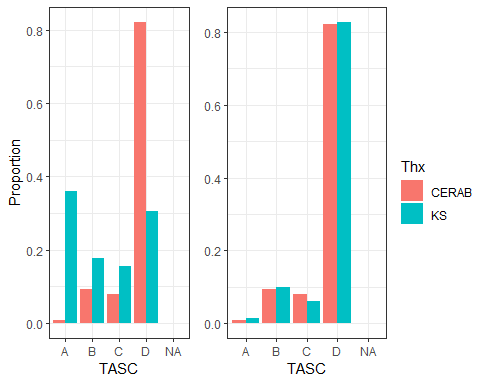


##
## [[2]]


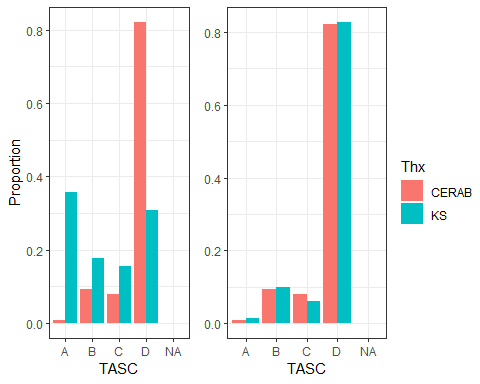


##
## [[3]]


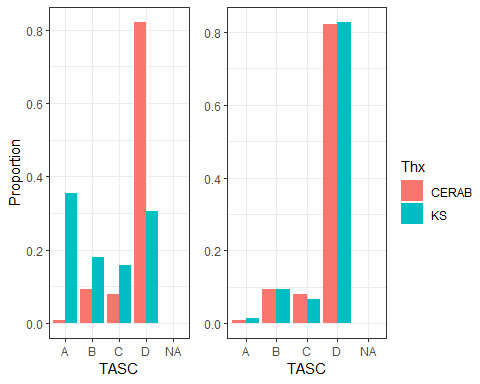


##
## [[4]]


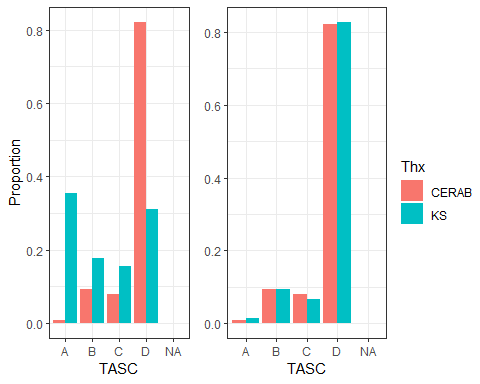


##
## [[5]]


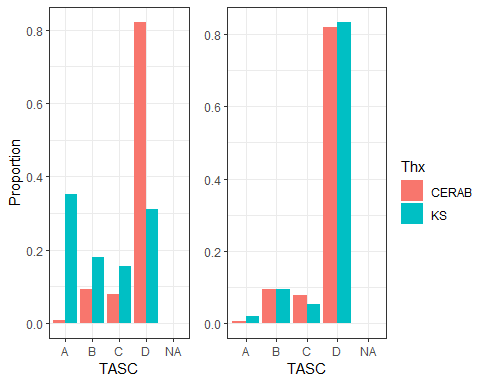


##
## [[6]]


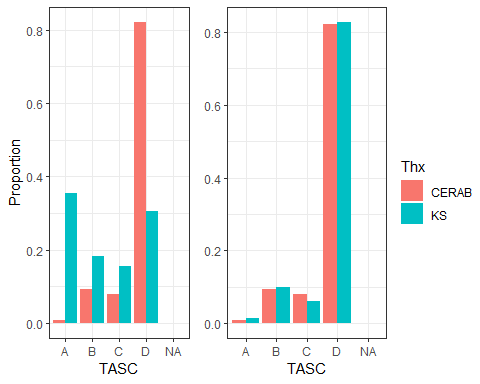


##
## [[7]]


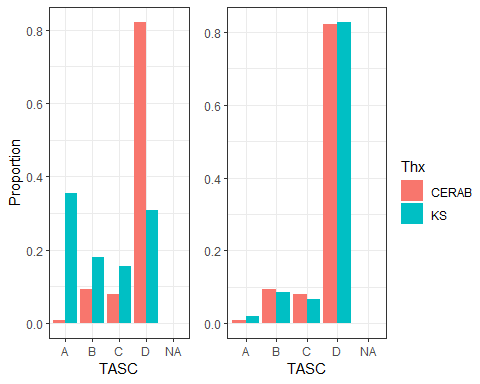


##
## [[8]]


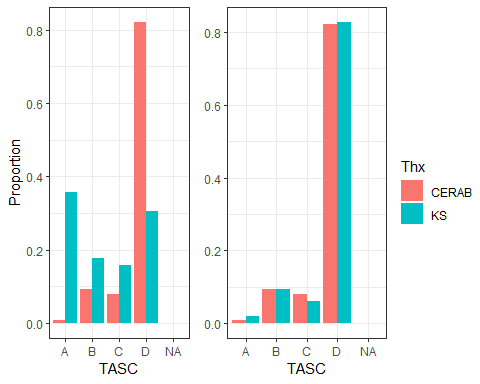


##
## [[9]]


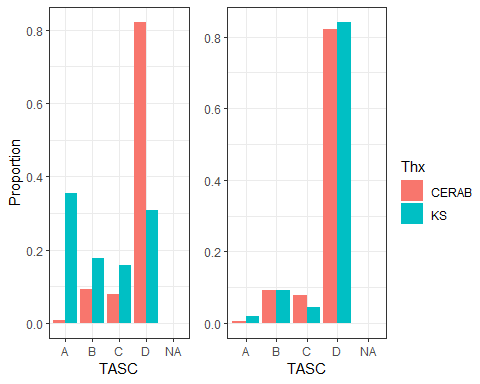


##
## [[10]]


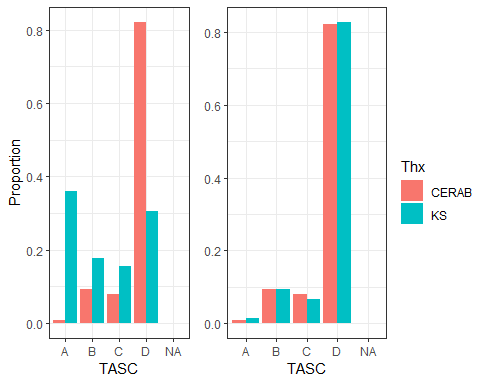


*Abbreviations: CERAB = Covered Endovascular Reconstruction of the Aorto-iliac Bifurcation; KS = Kissing Stents; Thx = treatment*

**Rutherford status before the procedure before (left) and after (right) matching in all ten imputed datasets**

## [[1]]


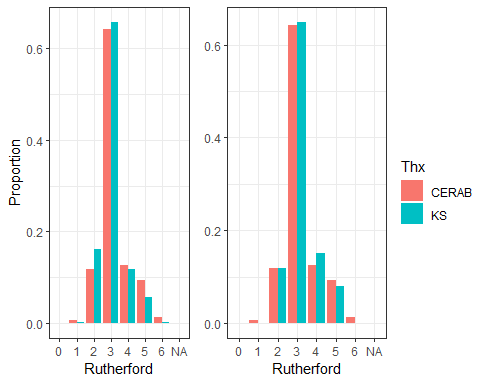


##
## [[2]]


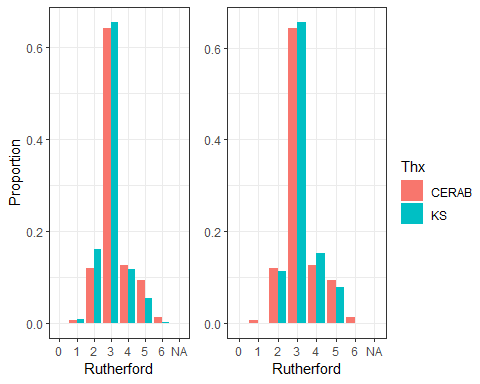


##
## [[3]]


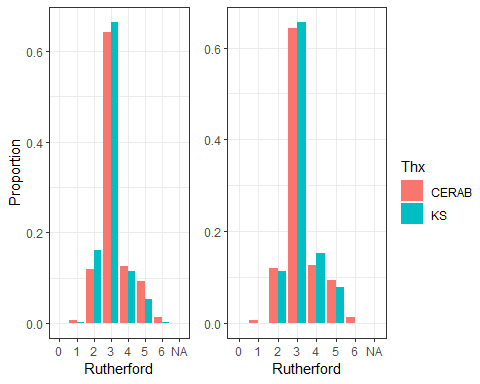


##
## [[4]]


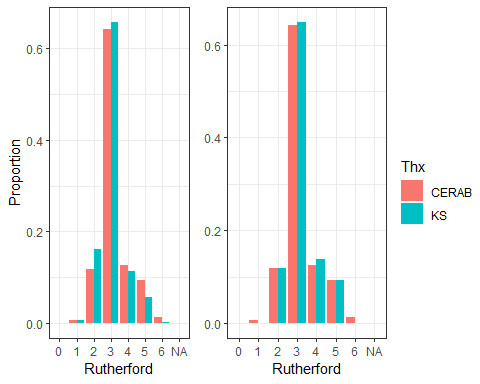


##
## [[5]]


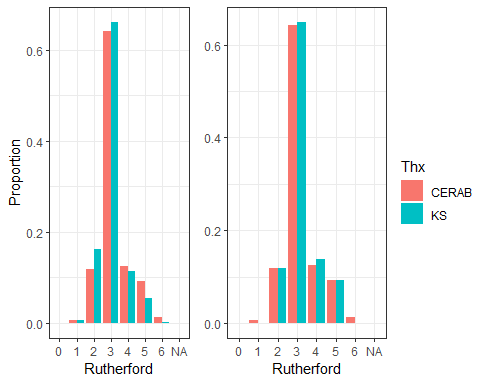


##
## [[6]]


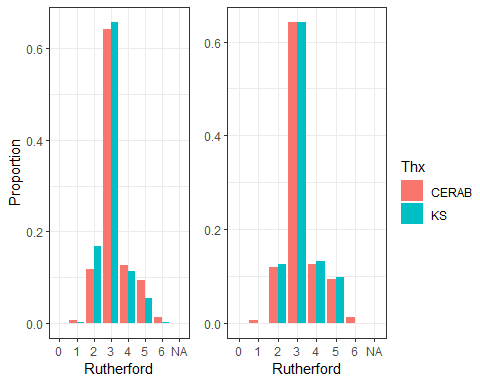


##
## [[7]]


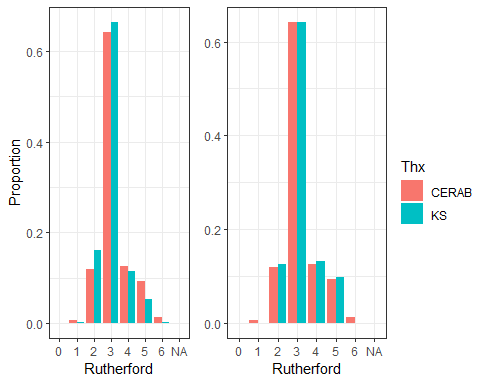


##
## [[8]]


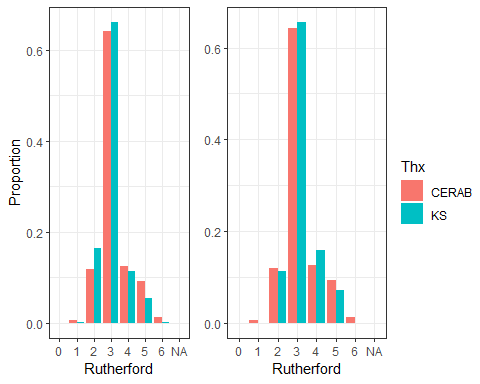


##
## [[9]]


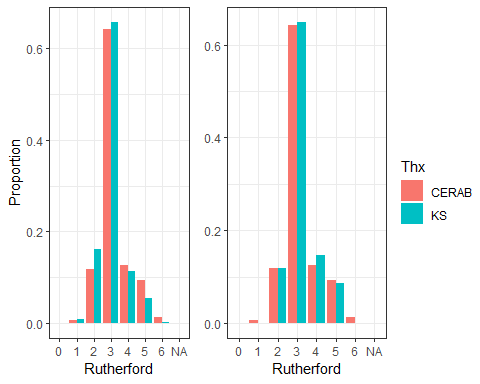


##
## [[10]]


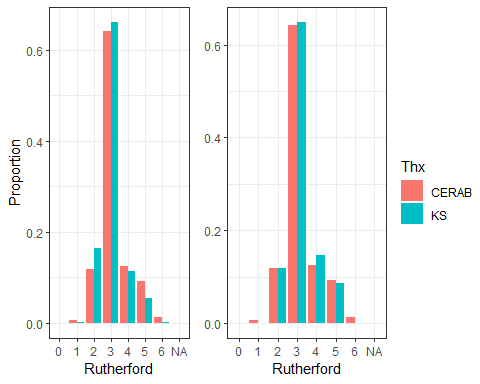


*Abbreviations: CERAB = Covered Endovascular Reconstruction of the Aorto-iliac Bifurcation; KS = Kissing Stents; Thx = treatment*

**Proportion of individuals with and without a previous intervention before (left) and after (right) matching in all ten imputed datasets**

## [[1]]


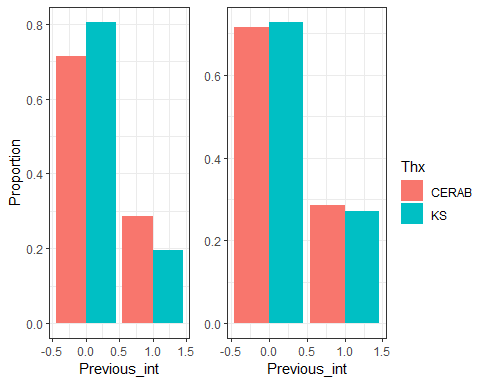


##
## [[2]]


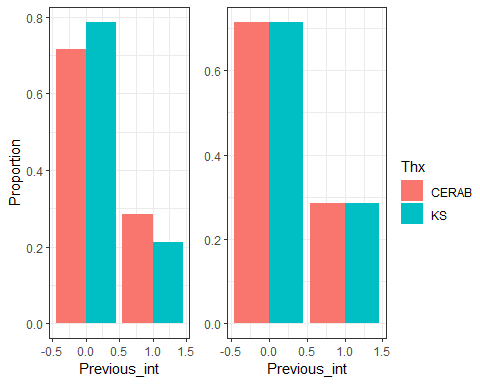


##
## [[3]]


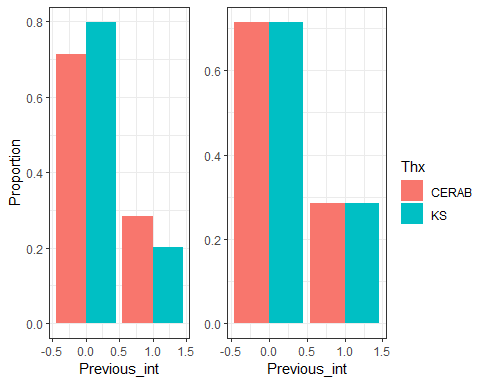


##
## [[4]]


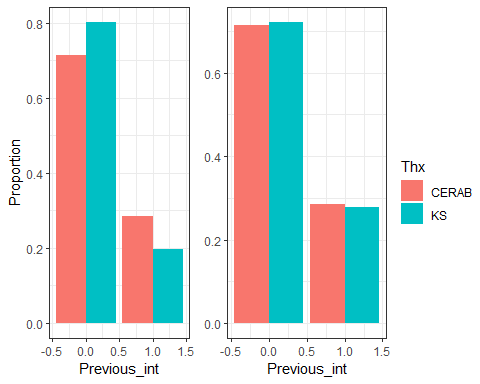


##
## [[5]]


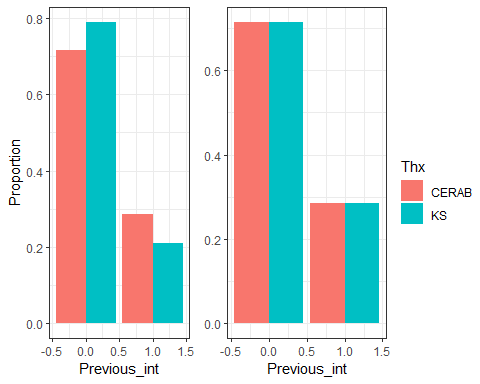


##
## [[6]]


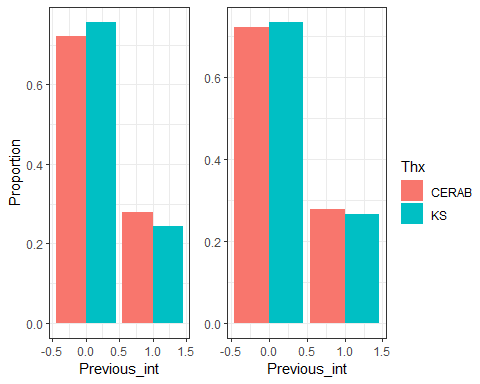


##
## [[7]]


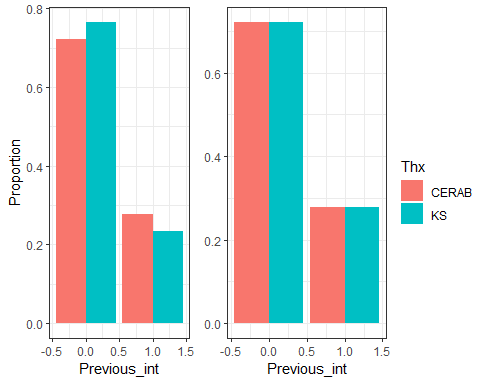


##
## [[8]]


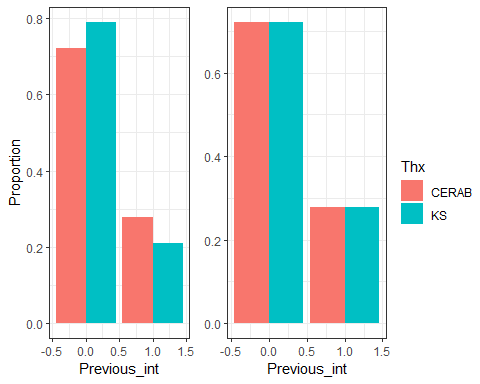


##
## [[9]]


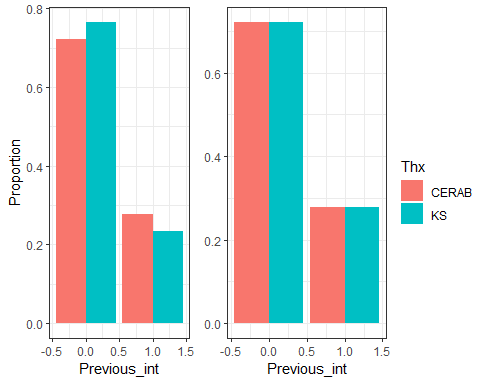


##
## [[10]]


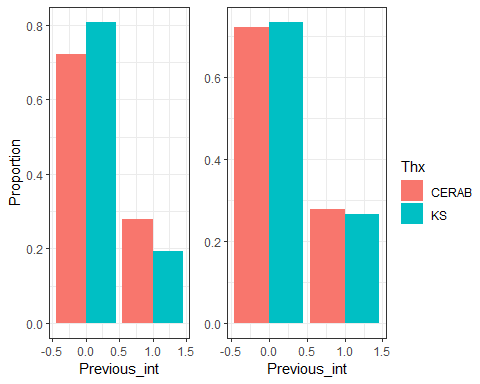


*Abbreviations: CERAB = Covered Endovascular Reconstruction of the Aorto-iliac Bifurcation; KS = Kissing Stents; Thx = treatment*

After matching, parametric survival distributions were fitted to the matched CERAB and KS groups separately to estimate the probability of reinterventions. The following parametric distributions were fitted to the reintervention time-to-event data of each imputed dataset: exponential, Weibull, Gompertz, lognormal, loglogistic, and gamma. Since we did not extrapolate beyond the observation period (5 years) of the time-to-event data, we selected the best fitting distribution using the Akaike Information criterion. This procedure was repeated in all imputed dataset. The best fitting distributions in all imputed datasets were the lognormal and the gompertz distributions.

## Probability of target lesion reintervention: Open repair versus CERAB

To estimate the probability of reintervention in the open repair strategy, a hazard ratio for the occurrence of reintervention in the open repair strategy versus CERAB was estimated using the guidelines for matching-adjusted indirect treatment comparison (MAIC) reported in the Technical Support Document 18 of the Decision Support Unit of the National Institute for Health and Care Excellence^6^. We performed an unanchored MAIC since we did not have randomised evidence concerning CERAB versus KS and Dorigo et al.^5^ is a non-randomised comparison of open repair versus KS.
In this MAIC, the CERAB individual-level data was re-weighted according to the aggregated data of individuals who underwent open repair in the study of Dorigo et al.^5^ This study was identified by searching the reference lists of recent systematic reviews and meta-analyses, known by the authors, focusing on open repair and endovascular revascularisation of AIOD^7,8^. Dorigo et al.^5^ was selected to perform the MAIC because it provided aggregated individual characteristics which were also available in the CERAB dataset and because it displayed a Kaplan-Meier curve of the probability of experiencing a reintervention in the open repair group.
To perform the MAIC, the Kaplan-Meier curve of the open repair group was digitised using the [PlotDigitizer Online App](https://plotdigitizer.com/app) and the individual-level data was reconstructed using the algorithm described in Rugola et al.^9^.
The CERAB datasets was re-weighted using the following characteristics: age, gender, Rutherford status, and TASC C or D before surgery. Since the standard deviation of the mean age of the open repair group was not reported in Dorigo et al.^5^, we assumed that the ratio between the standard deviation and the mean age of the CERAB dataset applied to the Dorigo et al. aggregated data^5^.
The following tables and figures illustrate the impact of the MAIC on the estimated hazard ratio of open repair versus CERAB for the occurrence of reinterventions. Only the results in the first imputed dataset are shown since the imputation did not affect the results of the MAIC.

1. The unmatched aggregated characteristics of the CERAB group used in the MAIC.

Unadjusted aggregated characteristics of CERAB and open repair group from Dorigo et al.

|  | CERAB | Open repair |
| --- | --- | --- |
| Mean.Age | 61.6 | 63.3 |
| SD.Age | 9.9 | 10.2 |
| Percentage.female | 49 % | 14% |
| Percentage.Rutherford.status.3 | 64 % | 65% |
| Percentage.Rutherford.status.4 | 13 % | 28% |
| Percentage.Rutherford.status.5 | 9 % | 5% |
| Percentage.Rutherford.status.6 | 1 % | 2% |
| Percentage.TASC.C.lesion | 8 % | 6% |
| Percentage.TASC.D.lesion | 82 % | 94% |

1. Matched aggregated CERAB data versus aggregated open repair data of Dorigo et al.

The table below shows that the MAIC increased the similarity in baseline characteristics between the matched CERAB group and the open repair group of Dorigo et al.

Adjusted aggregated characteristics of CERAB and open repair group from Dorigo et al.

|  | CERAB | Open repair |
| --- | --- | --- |
| Mean.Age | 63.3 | 63.3 |
| SD.Age | 10.3 | 10.2 |
| Percentage.female | 14 % | 14% |
| Percentage.Rutherford.status.3 | 65 % | 65% |
| Percentage.Rutherford.status.4 | 28 % | 28% |
| Percentage.Rutherford.status.5 | 5 % | 5% |
| Percentage.Rutherford.status.6 | 2 % | 2% |
| Percentage.TASC.C.lesion | 6 % | 6% |
| Percentage.TASC.D.lesion | 94 % | 94% |

1. Rescaled weights distribution and Effective Sample Size

The median rescaled weight was 0.4, with a range of 0 - 6.4. The number of individuals assigned with a weight of 0 was 0. The histogram of these rescaled weights shows that a substantial proportion of individuals were assigned a weight close to zero. Two individuals were assigned a weight above 4. Accordingly, the effective sample size of 65 indicates that weights were variable and that the estimated hazard ratio may be unstable.

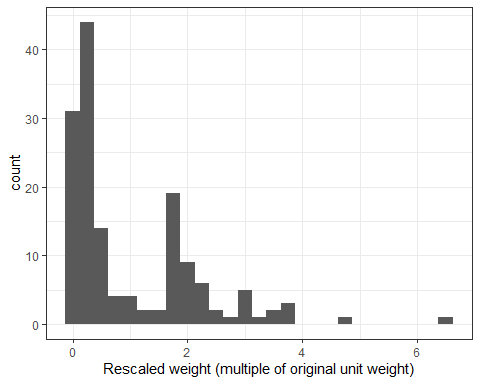
 . Estimated hazard ratio
The table and figure below shows the impact of matching on the estimated hazard ratio for the occurrence of reintervention of open repair versus CERAB. Matching did not markedly influence the point estimate of the comparison but increase the uncertainty surrounding this point estimate.

Estimated adjusted and unadjusted hazard ratio of open repair versus CERAB for the occurrence of reintervention

| Comparison_type | Estimate | Lower_bound_95%CI | Higher_bound_95%CI |
| --- | --- | --- | --- |
| NAIVE | 0.238 | 0.078 | 0.730 |
| MAIC | 0.285 | 0.078 | 1.044 |

1. Limitation and conclusion
   We were only able to adjust the comparison of open repair versus CERAB for a limited number of individuals’ characteristics. The distribution of the rescaled weights and the effective sample size indicate that the estimated hazard ratio may be unstable.

## Other transition probabilities

### Probability of amputation

No amputation was observed in the CERAB dataset, however these patients are at risk of experiencing an amputation of the lower limbs. Hence, the probability of amputation was obtained from an English study of individuals who had undergone CERAB for the treatment of AIOD^10^.

### Probability of disease-related death

The disease-related probability of death was estimated using the number of disease-related death and total follow-up time of individuals included in the CERAB dataset. This probability was assumed to be constant over time. The same disease-related probability of death was used in all strategies.

### Probability of death due to other causes

To avoid double counting, deaths due to peripheral arterial diseases were removed from the general population mortality. General population mortality rates from 2020 were used for this analysis^11^.

## Utility values

For the R0-3 and R4-6 health states, utility values were calculated as the weighted average of the proportion of individual having each Rutherford status at 6 weeks by the utility value assigned to each Rutherford status. Each Rutherford status was assigned a different utility value. Utility values were obtained from Chalmers et al.^12^ for Rutherford statuses 1 to 5. The utility value for Rutherford status 6 was obtained from^13^. Individuals with a Rutherford status of 0 were assumed to have a utility value equal to the Dutch general population^14^.
Explanation of the studies providing utility values:
- Chalmers et al.^12^ collected EQ-5D scores in 150 individuals with superior femoral artery occlusion or severe stenosis in the United Kingdom.
- De Vries et al.^13^ collected the EQ-5D-3L in 450 individuals suffering from peripheral artery disease (PAD).
- van Stel et al.^15^ estimated the impact of secondary cardiovascular event on quality of life using EQ-5D scores obtained during two Dutch trials including individuals suffering from PAD^16,17^. -^15^ provides gender-specific quality of life losses. These gender-specific disutility values were weighted by the proportion of male and female in the CERAB group and was assumed equal among the strategies.

## Resource use and costs

The costs of all procedures contained the costs of using the operating room and material costs (stents or graft). For CERAB and KS, the cost of the procedure was estimated using the procedure time registered in the CERAB database, and was assumed equal for CERAB and KS. Material costs were based on the number of stents reported in the CERAB and KS databases. For open repair, the time of the open repair procedure was calculated using the procedure time of 33 patients treated for abdominal aortic aneurysm at Rijnstate hospital. Material costs consisted of the graft costs.
For the CERAB and KS strategy, length of stay at the regular ward and at the ICU, and the probability of being admitted at the ICU were estimated using the CERAB database. For the open repair strategy, length of stay at the hospital and ICU were based on literature^18^.

Assumptions made concerning the costs of the procedures:

- We assumed that the same type of stents was used for CERAB (iliacal stents) and KS.
- A minimum of three stents was used for CERAB (one for the aorta, two iliacal) and a minimum of two stents was used to perform a KS procedure.
- To determine the costs of the KS stents, we assumed that 50% of the stents were covered stents and 50% were uncovered.
- The hourly costs of a regular operating room and the prices of the stents and graft were obtained from the hospital gathering evidence on CERAB
- Procedural complications led to longer procedure time, which was calculated based on the CERAB individual-level data. The procedure time calculations was thus stratified by whether a procedural complication occurred and was assumed equal between the CERAB and KS because both are endovascular procedures.
- For the CERAB and KS strategy, length of stay at the regular ward was estimated using the CERAB individual-level data and was stratified for the occurrence of post-procedural complications. Hence, post-procedural complications led to longer hospitalisation.
- Since solely summary data was available concerning the open repair procedure time, it was assumed that the mean (and standard deviation) time of the procedure contains procedures with and without procedural complications.

## Model inputs overview

The following tables contain parameter values according to the distribution used to parameterise the parameters during the probabilistic analysis.

Parameter values of lognormal survival models (9 imputed datasets) for the occurrence of reintervention

| Description | Mean | SD | Distribution | Source |
| --- | --- | --- | --- | --- |
| Meanlog (natural scale) CERAB survival model of 9 multiply imputed datasets | 6.929 | 0.868 | CHOLESKY | CERAB dataset |
| SDlog (natural scale) CERAB survival model of 9 multiply imputed datasets | 1.210 | 0.182 | CHOLESKY | CERAB dataset |
| Meanlog (natural scale) KS survival model | 5.219 | 0.517 | CHOLESKY | KS dataset |
| SDlog (natural scale) KS survival model | 1.087 | 0.138 | CHOLESKY | KS dataset |
| Meanlog (natural scale) KS survival model | 5.572 | 0.566 | CHOLESKY | KS dataset |
| SDlog (natural scale) KS survival model | 1.126 | 0.143 | CHOLESKY | KS dataset |
| Meanlog (natural scale) KS survival model | 5.981 | 0.623 | CHOLESKY | KS dataset |
| SDlog (natural scale) KS survival model | 1.183 | 0.149 | CHOLESKY | KS dataset |
| Meanlog (natural scale) KS survival model | 6.634 | 0.822 | CHOLESKY | KS dataset |
| SDlog (natural scale) KS survival model | 1.260 | 0.170 | CHOLESKY | KS dataset |
| Meanlog (natural scale) KS survival model | 6.071 | 0.674 | CHOLESKY | KS dataset |
| SDlog (natural scale) KS survival model | 1.165 | 0.157 | CHOLESKY | KS dataset |
| Meanlog (natural scale) KS survival model | 6.113 | 0.710 | CHOLESKY | KS dataset |
| SDlog (natural scale) KS survival model | 1.304 | 0.151 | CHOLESKY | KS dataset |
| Meanlog (natural scale) KS survival model | 6.260 | 0.716 | CHOLESKY | KS dataset |
| SDlog (natural scale) KS survival model | 1.317 | 0.150 | CHOLESKY | KS dataset |
| Meanlog (natural scale) KS survival model | 7.288 | 0.987 | CHOLESKY | KS dataset |
| SDlog (natural scale) KS survival model | 1.349 | 0.183 | CHOLESKY | KS dataset |
| Meanlog (natural scale) KS survival model | 6.477 | 0.741 | CHOLESKY | KS dataset |
| SDlog (natural scale) KS survival model | 1.242 | 0.161 | CHOLESKY | KS dataset |

Parameter values of Gompertz survival models (1 imputed dataset) for the occurrence of reintervention

| Description | Mean | SD | Distribution | Source |
| --- | --- | --- | --- | --- |
| Shape (natural scale) CERAB survival model of 1 multiply imputed dataset | -0.042 | 0.018 | CHOLESKY | CERAB dataset |
| Rate (natural scale) CERAB survival model of 1 multiply imputed dataset | -4.608 | 0.331 | CHOLESKY | CERAB dataset |
| Shape (natural scale) KS survival model | -0.106 | 0.031 | CHOLESKY | KS dataset |
| Rate (natural scale) KS survival model | -3.875 | 0.305 | CHOLESKY | KS dataset |

Table: Parameter values of the discrete-time Gompertz survival models for progression and regression in Rutherford status

|Description | Mean| SD$\left\vert Source \right\vert\left\vert:----------------------------------------------------------------- \right\vert------:\left\vert-----: \right\vert:-------------\left\vert\right\vert InterceptoftheGompertzmodeltoestimateP\left( progression \right)$$ | -4.016| 0.690|CERAB dataset | |Time parameter of the Gompertz model to estimate P(progression)

$$\left| -0.238 \right|0.182\left| CERABdataset \right||InterceptoftheGompertzmodeltoestimateP\left( regression \right)$$

| -1.494| 1.104|CERAB dataset | |Time parameter of the Gompertz model to estimate P(regression)$$ | 0.142| 0.442|CERAB dataset |

Parameter values of the (truncated) normal distributions

| Description | Mean | SD$ | Number of observations | Distribution | Source |
| --- | --- | --- | --- | --- | --- |
| Log(hazard ratio) reintervention Open repair versus CERAB - MAIC | -1.255 | 0.662 | NA | NORMAL | ^5^ and ITC |
|  | -0.642 | 0.285 | NA | NORMAL |  |
| Log(hazard ratio) reintervention Open repair versus CERAB - NAIVE | -1.435 | 0.572 | NA | NORMAL | ^5^ and ITC |
|  | -0.237 | 0.397 | NA | NORMAL |  |
| Log(odds ratio) reintervention Open repair versus CERAB - MAIC | -0.712 | 0.583 | NA | NORMAL | ^5^ and ITC |
| Log(odds ratio) reintervention Open repair versus CERAB - NAIVE | -0.880 | 0.520 | NA | NORMAL | ^5^ and ITC |
| Log(odds ratio) reintervention Open repair versus CERAB - STC | -0.863 | 0.544 | NA | NORMAL | ^5^ and ITC |
| KS - monthly rate of reintervention | 0.010 | 0.002 | NA | NORMAL | Matched KS datasets |
| Disutility value: Major amputation men | 0.161 | 0.170 | NA | NORMAL | ^15^ |
| Disutility value: Major amputation women | 0.335 | 0.218 | NA | NORMAL | ^15^ |
| Utility value: Post-amputation disutility men | -0.057 | 0.186 | NA | NORMAL | ^15^ |
| Utility value: Post-amputation disutility women | 0.137 | 0.070 | NA | NORMAL | ^15^ |
| Number of stents when more than 2 are used during KS | 2.700 | NA | 2# | TRUNCATED NORMAL | ^5^ |
| Open repair - Operating room time in minutes | 251.000 | 96.296 | 9257 | NORMAL | ^19^ |
| Health state costs: Post amputation first six months | 2796.137 | NA | NA | NORMAL | ^20^ |
| HR reintervention CERAB vs KS (weighted) | -0.215 | 0.408 | NA | NORMAL | CERAB & KS dataset |
| HR reintervention CERAB vs KS (matched) | -0.343 | 0.361 | NA | NORMAL | CERAB & KS dataset |

Parameter values of the parameters with fixed values

| Description | Mean | Source |
| --- | --- | --- |
| Proportion woman in CERAB dataset | 0.497 | CERAB dataset |
| Utility value: Dead | 0.000 | Assumption |
| Number of stent used for KS operation | 2.000 | Expert opinion |
| Costs of antibiotica treatment | 1990.218 | Internal price Rijnstate |
| Daily costs ascal | 0.060 | Medicijnkosten.nl |
| Daily costs clopidogrel | 0.050 | Medicijnkosten.nl |
| Health state costs: Dead | 0.000 | Assumption |
| Price policlinic visit | 97.730 | Dutch guidelines for health economic evaluations |
| Costs of an embolectomy | 2599.741 | Internal price Rijnstate - zSuperB trial 2019 document |
| Costs of treating a hernia - laparoscopy | 3612.000 | Internal price Rijnstate |
| Costs of treating a hernia - open surgery | 2791.000 | Internal price Rijnstate |
| Costs hospital day | 496.269 | Internal price - 20220309_kosten_CERAB_KS_open_Rijnstate_deels_vertrouwelijk |
| Costs hybrid operating room per hour | 1281.041 | Internal price - 20220309_kosten_CERAB_KS_open_Rijnstate_deels_vertrouwelijk |
| Costs ICU day | 2958.099 | Internal price - 20220309_kosten_CERAB_KS_open_Rijnstate_deels_vertrouwelijk |
| Costs of treating an ileus | 2256.140 | Internal price Rijnstate |
| Costs MCU day | 2109.862 | Internal price - 20220309_kosten_CERAB_KS_open_Rijnstate_deels_vertrouwelijk |
| Costs regular operating room per hour | 287.847 | Internal price - 20220309_kosten_CERAB_KS_open_Rijnstate_deels_vertrouwelijk |
| Price policlinic visit | 111.168 | Dutch guidelines for health economic evaluations |
| Costs of a PTA | 427.041 | Internal price Rijnstate - zSuperB trial 2019 document |
| Daily costs statines | 0.040 | Medicijnkosten.nl |
| Costs per stent (CERAB) - aortic | 2258.953 | Rijnstate_intern - 20201028 Prijzen ivm research |
| Costs per stent (CERAB) - iliac | 1694.214 | Rijnstate_intern - 20201028 Prijzen ivm research |
| Costs per stent (KS) | 915.548 | Rijnstate_intern - 20201028 Prijzen ivm research |
| Costs of an thrombolysis | 778.188 | Internal price Rijnstate - zSuperB trial 2019 document |
| CERAB dataset | 61.650 | mean_age |

Parameter values of beta distributions parameterised with mean and standard error using the method of moment

| Description | Mean | SD$ | Number of observations | Source |
| --- | --- | --- | --- | --- |
| CERAB, KS, and OSR - Monthly probability of dying from the disease | 0.003 | 0.001 | 156 | CERAB dataset |
| Utility value general population:50-59 | 0.857 | 0.183 | 186 | ^14^ |
| Utility value general population:60-69 | 0.839 | 0.179 | 158 | ^14^ |
| Utility value general population:70+ | 0.852 | 0.148 | 106 | ^14^ |
| Utility value: Rutherford status 1 | 0.550 | 0.250 | 16 | ^12^ |
| Utility value: Rutherford status 2 | 0.430 | 0.300 | 56 | ^12^ |
| Utility value: Rutherford status 3 | 0.360 | 0.310 | 42 | ^12^ |
| Utility value: Rutherford status 4 | 0.270 | 0.280 | 8 | ^12^ |
| Utility value: Rutherford status 5 | 0.380 | 0.380 | 17 | ^12^ |
| Utility value: Rutherford status 6 | 0.310 | NA | NA | ^13^ |

Parameter values of beta distributions parameterised with number of events and observations

| Description | Mean | Number of events | Number of observations | Source |
| --- | --- | --- | --- | --- |
| CERAB - Probability of amputation | <0.001 | 2.000 | 116 | ^10^ |
| KS - Probability of amputation | <0.001 | 2.000 | 116 | ^10^ |
| Probability of ICU & MCU admission in the CERAB strategy | 0.02 | 3.000 | 151 | CERAB dataset |
| Probability of ICU & MCU admission in the CERAB strategy | 0.02 | 3.000 | 151 | CERAB dataset |
| Probability of ICU admission in the CERAB strategy | 0.02 | 3.000 | 151 | CERAB dataset |
| Probability of ICU admission in the KS strategy | 0.02 | 3.000 | 151 | KS dataset |
| Probability of MCU admission in the CERAB strategy | 0.146 | 22.000 | 151 | CERAB dataset |
| Probability of MCU admission in the CERAB strategy | 0.146 | 22.000 | 151 | CERAB dataset |
| Probability of post-procedural complications in the CERAB strategy | 0.205 | 31.000 | 151 | CERAB dataset |
| Probability of post-procedural complications in the KS strategy | 0.205 | 31.000 | 151 | KS dataset |
| Open repair: probability of post-procedure complications | 0.207 | 17.000 | 82 | ^5^ |
| Probability of procedural complication in the CERAB strategy | 0.126 | 19.000 | 151 | CERAB dataset |
| Probability of procedural complication in the KS strategy | 0.126 | 19.000 | 151 | KS dataset |
| Open repair: probability of procedure-related complications | 0.061 | 5.000 | 82 | ^5^ |
| Probability of procedure-related death | 0.006 | 1.000 | 157 | CERAB dataset; Assumption |
| Probability of death in KS strategy | 0.006 | 1.000 | 157 | CERAB dataset; Assumption |
| Open repair: probability of procedure-related death | 0.027 | 45.000 | 1679 | ^8^ |
| CERAB & KS: Probability of reintervention if PP is lost | 0.486 | 18.000 | 37 | CERAB dataset |
| CERAB - Probability to be in rutherford status 0-3 after the intervention  $\left\vert0.993 \right\vert0.007\left\vert151 \right\vert CERABdataset\left\vert\right\vert KS-Probabilitytobeinrutherfordstatus0-3aftertheintervention$ | 0.993 | 0.007 | 151 | CERAB dataset |
| CERAB & KS - Probability of technical success | 0.968 | 151.000 | 156 | CERAB dataset |
| CERAB & KS - Probability of technical success | 0.968 | 151.000 | 156 | CERAB dataset |
| Open repair: probability of undergoing an open repair of a hernia | 0.483 | 185.000 | 383 | Internal data |
| Probability of having more than 2 stents for KS | 0.344 | 44.000 | 128 | ^5^ |

Parameter values of gamma distributions parameterised using parameters from literature and method of moments

| Description | Mean | SD$ | Number of observations | Source |
| --- | --- | --- | --- | --- |
| Disutility value: Post-reintervention for men | 0.121 | 0.042 | NA | ^15^ |
| Disutility value: Post-reintervention for women | 0.105 | 0.057 | NA | ^15^ |
| Disutility value: Reintervention for men | 0.113 | 0.039 | NA | ^15^ |
| Disutility value: Reintervention for women | 0.117 | 0.083 | NA | ^15^ |
| Open repair - length of stay laparoscopic reconstruction hernia | 3.850 | 7.040 | 684.000 | Rijnstate - internal |
| Open repair - length of stay open reconstruction hernia | 6.970 | 13.650 | 976.000 | Rijnstate - internal |
| Open repair - number of hospital days | 7.760 | 2.105 | NA | ^8^ |
| Open repair - length of stay at the ICU | 5.300 | 3.900 | 30.000 | ^18^ |
| CERAB - Number of MCU days | 1.286 | 6.361 | 4.947 | CERAB dataset |
| Health state costs: Major amputation | 2628.166 | NA | NA | Rijnstate_intern |
| Costs material open repair surgery | 403.709 | 159.708 | NA | Internal price |
| Health state costs: Post amputation after first six months | 1398.068 | NA | NA | ^20^ |

Parameter values of gamma distributions parameterised through fitting to available data

| Description | Mean | Shape | Rate | Source |
| --- | --- | --- | --- | --- |
| Number of hospital days, without post-procedural complications | 2 | 2.000 | 0.782 | CERAB dataset |
| Number of hospital days, with post-procedural complications | 8 | 0.983 | 0.128 | CERAB dataset |
| CERAB - Number of ICU days | 8 | 0.877 | 0.115 | CERAB dataset |
| Surgery time (in minutes), without procedural complications | 109 | 3.000 | 0.026 | CERAB dataset |
| Surgery time (in minutes), with procedural complications | 176 | 3.000 | 0.019 | CERAB dataset |
| Number of stent used for CERAB operation | 4 | 9.000 | 2.000 | CERAB dataset |

Parameter values of dirichlet distributions

| Description | Mean | Grouping name | Source |
| --- | --- | --- | --- |
| Proportion of individuals with rutherford status 0 after CERAB intervention  $\left\vert0.638 \right\vert DIRICHLET_{0_{3}}\left\vert CERABdataset \right\vert\vert Proportionofindividualswithrutherfordstatus1afterCERABintervention$ | 0.202 | DIRICHLET_0_3 | CERAB dataset |
| Proportion of individuals with rutherford status 2 after CERAB intervention  $\left\vert0.084 \right\vert DIRICHLET_{0_{3}}\left\vert CERABdataset \right\vert\vert Proportionofindividualswithrutherfordstatus3afterCERABintervention$ | 0.076 | DIRICHLET_0_3 | CERAB dataset |
| Proportion of individuals with rutherford status 0 before CERAB intervention | 0.000 | DIRICHLET_0_6 | CERAB dataset |
| Proportion of individuals with rutherford status 0 before CERAB intervention | 0.006 | DIRICHLET_0_6 | CERAB dataset |
| Proportion of individuals with rutherford status 0 before CERAB intervention | 0.115 | DIRICHLET_0_6 | CERAB dataset |
| Proportion of individuals with rutherford status 0 before CERAB intervention | 0.637 | DIRICHLET_0_6 | CERAB dataset |
| Proportion of individuals with rutherford status 0 before CERAB intervention | 0.127 | DIRICHLET_0_6 | CERAB dataset |
| Proportion of individuals with rutherford status 0 before CERAB intervention | 0.102 | DIRICHLET_0_6 | CERAB dataset |
| Proportion of individuals with rutherford status 0 before CERAB intervention | 0.013 | DIRICHLET_0_6 | CERAB dataset |
| Proportion of individuals with rutherford status 4 after CERAB intervention  $\left\vert0.402 \right\vert DIRICHLET_{4_{6}}\left\vert CERABdataset \right\vert\vert Proportionofindividualswithrutherfordstatus5afterCERABintervention$ | 0.598 | DIRICHLET_4_6 | CERAB dataset |
| Proportion of individuals with rutherford status 6 after CERAB intervention$$ | 0.000 | DIRICHLET_4_6 | CERAB dataset |
| Probability cross-over after CERAB technical failure | 0.400 | DIRICHLET_FAIL_CERAB | CERAB dataset |
| Probability open-repair after CERAB technical failure | 0.200 | DIRICHLET_FAIL_CERAB | CERAB dataset |
| Probability watchful waiting after CERAB technical failure | 0.400 | DIRICHLET_FAIL_CERAB | CERAB dataset |
| Probability of undergoing a bypass as reintervention | 0.067 | DIRICHLET_REINT | CERAB dataset |
| Probability of undergoing an embolectomy as reintervention | 0.200 | DIRICHLET_REINT | CERAB dataset |
| Probability of undergoing a PTA as reintervention | 0.333 | DIRICHLET_REINT | CERAB dataset |
| Probability of undergoing a PTA + stent as reintervention | 0.133 | DIRICHLET_REINT | CERAB dataset |
| Probability of undergoing a PTA + thrombolysis as reintervention | 0.067 | DIRICHLET_REINT | CERAB dataset |
| Probability of undergoing an thrombolysis as reintervention | 0.200 | DIRICHLET_REINT | CERAB dataset |
| Open repair: probability of undergoing an infection treated through antibiotica as reintervention | 0.033 | DIRICHLET_REINT_OPEN | ^21^ |
| Open repair: probability of undergoing a treatment for hernia as reintervention | 0.633 | DIRICHLET_REINT_OPEN | ^21^ |
| Open repair: probability of undergoing a treatment for ileus as reintervention | 0.133 | DIRICHLET_REINT_OPEN | ^21^ |
| Open repair: probability of undergoing a thrombolysis as reintervention | 0.200 | DIRICHLET_REINT_OPEN | ^21^ |

*Abbreviations: CERAB = Covered Endovascular Reconstruction of the Aortic Bifurcation; KS = Kissing Stent; OR = Odds ratio* *Legend: $ In all tables, the standard deviation is presented if there is also a value for the “Number of observations”. These values have then been used to calculate the standard error of the parameter. If there is no value provided in the “Number of observation” column, then the standard error is presented. If there is no value for both variable, then a standard variation of 25% has been used for the standard error. $$The parameters of the distributions used for these inputs were influenced by the multiple imputation. The mean and standard deviation presented in this table were thus calculated based on the probabilistic parameters (since presenting all parameters obtained from the multiple imputation is not feasible)* *#This is the minimum value of the truncated normal distribution*

## Probabilistic analysis

The probabilistic analysis was performed using a Monte Carlo simulation. The distributions assigned to the model inputs are provided in the table above. Since we multiply imputed missing data ten times and that the multiple imputation influenced the matching results and the survival analysis of the KS group, we randomly selected one of the ten fitted survival distribution for each iteration of the probabilistic analysis and varied the parameter value of the survival distribution using a Cholesky decomposition. Additionally, to ensure that utility values remained in a logical order during the probabilistic analysis, we used the same random percentile for all beta distributions assigned to the utility values.

## Probabilistic one-way sensitivity analyses

Probabilistic one-way sensitivity analyses (POSA) were performed according to the descriptions of Vreman et al.^22^ and McCabe et al.^23^. Each input parameter of the model were in turn, fixed to their 2.5^th^, 12.5^th^, 25^th^, 75^th^, 87.5^th^, and 97.5^th^ percentiles and a probabilistic analysis was performed (1,000 iterations per multiply imputed dataset). The incremental costs and QALYs of open repair and CERAB versus KS were then recorded for each of these POSAs. The mean incremental net monetary benefits, using a €50,000 per QALY willingness-to-pay threshold were then calculated for each POSA (the incremental net monetary benefits are calculated by multiplying the incremental QALYs by the willingness-to-pay threshold and then subtracting the incremental costs). For clarity, the results were plotted in a spider plot for Open repair and CERAB separately.

## Subgroup analyses

The method to estimate the inputs for the subgroup analyses were the same as for the basecase analysis, except that the HR of open repair versus CERAB for the occurrence of reintervention could not be re-estimated in each subgroup. Hence, it was assumed that this HR was equal to the basecase analysis in all subgroups.

## Health economic model validation

The health economic model was validated using the TECH-VER and the AdviSHE checklists.

### Part A: Validation of the conceptual model

#### A1/ Face validity testing (conceptual model)

Have experts been asked to judge the appropriateness of the conceptual model?
Yes, Prof. Reijnen has been actively involved in the conceptualisation fo the HE model. Prof. Reijnen can be considered a clinical expert in this field, since he developed and performs research on the effectiveness of the CERAB procedure.
Prof. Reijnen first suggested to conceptualise the HE model using patency, however, he recognised that using patency as a measure of effectiveness was not practical to define homogeneous health states. He further mentioned that only individuals with Rutherford status 4-6 could suffer an amputation. He further argued that only individuals with Rutherford status 4-6 could experience a reintervention. However, we could not implement a Rutherford-status dependent probability of experiencing a reintervention due to the limited number of reintervention in de CERAB database and the paucity of evidence concerning the probability of reintervention per Rutherford status in the KS database and literature relating to open repair.

#### A2/ Cross validity testing (conceptual model)

The current conceptual model has not been compared to other health economic models due to the absence of health economic evaluations focusing on a similar decision problem.

### Part B: Input data validation

#### B1/ Face validity testing (input data)

Prof. Reijnen has been consulted during the development of the HE model to face validate the input data. For instance, the probability of experiencing a reintervention in the CERAB and KS strategies were estimated through other methods, which led to implausibly high probabilities of reintervention. Hence calculation were updated.
In addition, financial employees have been consulted to obtain prices of resource use consumed at the hospital.

#### B2/ Model fit testing

The Akaike Information Criterion had been used to selected the best fitting survival curve used to estimate the probability of reintervention the CERAB strategy.

### Part C: Validation of the computerized model

#### C1/ External review

The HE model has not been externally reviewed.

#### C2/ Extreme value testing

Described under “Technical validation” paragraph.

#### C3/ Testing of traces

Described under “Technical validation” paragraph.

#### C4/ Unit testing

Described under “Technical validation” paragraph.

#### Technical validation

Validation of probabilistic model inputs was performed using the R package testthat 3.2.1^24^. The following tests were performed on model the probabilistic model inputs:
- all probabilities are greater than or equal to 0?
- all probabilities are lower than or equal to 1?
- all utilities are greater than or equal to 0? - all utilities are lower than or equal to 1?
- all costs are greater than or equal to 0?
- all resource use are greater than or equal to 0?
- all hazard ratios are greater than 0?
The following tests were performed to investigate whether the health economic model behaved as intended. These tests were applied to the probabilistic results if not mentioned otherwise. We tested that:
- the sum transition probabilities if the decision tree equals 1 in all strategies (deterministic only)
- discounted outcomes were lower than undiscounted outcomes
- discounted outcomes equal undiscounted outcomes if discount rates are set to 0
- total QALYs equal total LYs if utility values are set to 1 and disutility values are set to 0
- total QALYs equal 0 if utility and disutility values are set to 0
- total costs equal 0 if costs inputs are set to 0
- all cycles contain 1,000 individuals (deterministic results only)
- the number of individuals is greater or equal to 0 in all health states and cycles (deterministic results only)
- the number of dead individuals increases over cycles (deterministic results only)
- simulated individuals did not die if probabilities of death are set to 0 (deterministic results only)
- setting probabilities to the same values between strategies (CERAB and KS) results in same LYs and QALYs
- number of reintervention is always lower (or equal) in the open repair strategy than in the CERAB and KS strategy
All these tests were successfully passed.
Additionally, warnings were implemented in the health economic model to debug the script if it led to incorrect behaviours, for instance when the sum of probabilities relating to a health state exceeded one.

### Part D: Operational validation

#### D1/ Face validity testing (model outcomes)

The following outcomes were face validated with Prof. Reijnen: - The 5-year probability of experiencing a reintervention in each strategy
The probability of experiencing a reintervention in the CERAB strategy was considered plausible, while the probability of experiencing a reintervention in the open repair group was considered at the lower end of probabilities mentioned in the literature. Hence, this was considered to be a conservative assumption.
- The 5-year mortality
The 5-year mortality in the CERAB and KS strategies based on the HE model was 22 %, and it was 24 % for the open repair group. These estimates were considered plausible by Prof. Reijnen.
- The ranking of the strategies concerning their total health outcomes and costs

#### D2/ Cross validation testing (model outcomes)

The outcomes of the current HE model have not been cross-validated versus the outcomes of another HE model since we are not aware of any previous HE analysis focusing on a similar decision problem.

#### D3/ Validation against outcomes using alternative input data

We did not perform such validation check because we are not aware of previous health economic analyses focusing on a similar decision problem.

#### D4/ Validation against empirical data

##### D4.A/ Comparison against the data sources on which the model is based (dependent validation).

The 5-year probability of experiencing a reintervention in the CERAB strategy was 73 % based on the individual-level data and 17 % (11% - 27%) in the HE model, which are similar estimates.

##### D4.B/ Comparison against a data source that was not used to build the model (independent validation).

The table below provides a comparison of the probability of reintervention estimated through the HE model versus estimates provided in the literature. The discrepancy between the HE model estimation and Saratzis et al.^10^ estimate at 42 months is probably explained by the difference in outcomes for which the estimates were calculated: Saratzis et al.^10^ focused on target-lesion revascularisation while we focused on clinically-driven target-lesion revascularisation. We however estimated higher reintervention rates for the KS group compared with Piffaretti et al.^25^ and Dorigo et al.^5^. The observed differences with Piffareti et al. may be explained by the different TASC classification of individuals included in Piffareti compared to the matched KS group used in the current study.

| Study first author | Reference | Strategy | Outcome study | Numeric value | Outcome HE model (probability of reintervention) |
| --- | --- | --- | --- | --- | --- |
| Saratzis et al. | ^10^ | CERAB | 1-y freedom TLR | 94% (95% CI 90.4% to 98.3%) | 7 % (5 %-11 %) |
| Saratzis et al. | ^10^ | CERAB | 42months freedom TLR | 70-75%^a^ | 15 % (9 %-23 %) |
| Piffaretti et al. | ^25^ | Endo | 1-y freedom from reintervention | 98% (95% CI 96% to 99%) | 13 % (7 %-22 %)^b^ |
| Piffaretti et al. | ^25^ | Endo | 2-y freedom from reintervention | 97% (95% CI 94% to 98.5%) | 20 % (11 %-32 %)^b^ |
| Dorigo et al. | ^5^ | KS | 6-y freedom from reintervention | 89% (SE: 0.04) | 29 % (17 %-46 %)^c^ |

^a^ read from the Kaplan-Meier curve by XP.
^b^ KS estimates
^c^ 5 year KS estimates
*Abbreviations: CI = confidence interval; Endo = endovascular; HE = health economic; KS = Kissing Stent| TLR = target lesion reintervention; y = year.*

### Part E: Other validation techniques

No additional validation techniques have been used.

## Determining the willingness to pay threshold

To determine the willingness-to-pay threshold to use in the current study, we have use the total discounted QALY gained in the KS strategy from the scenario analysis using a lifetime time horizon. These outcomes were used to populate the online [iDBC tool](https://www.imta.nl/tools/idbc/)^26^, which resulted in a disease burden of 0.52. This disease burden correspond to a willingness-to-pay threshold of €50,000.

# Additional results

## Cost effectiveness plane

The Figure below presents the cost effectiveness plane for all strategies. As can be seen, open repair leads to the highest costs and least QALYs compared to CERAB and KS.


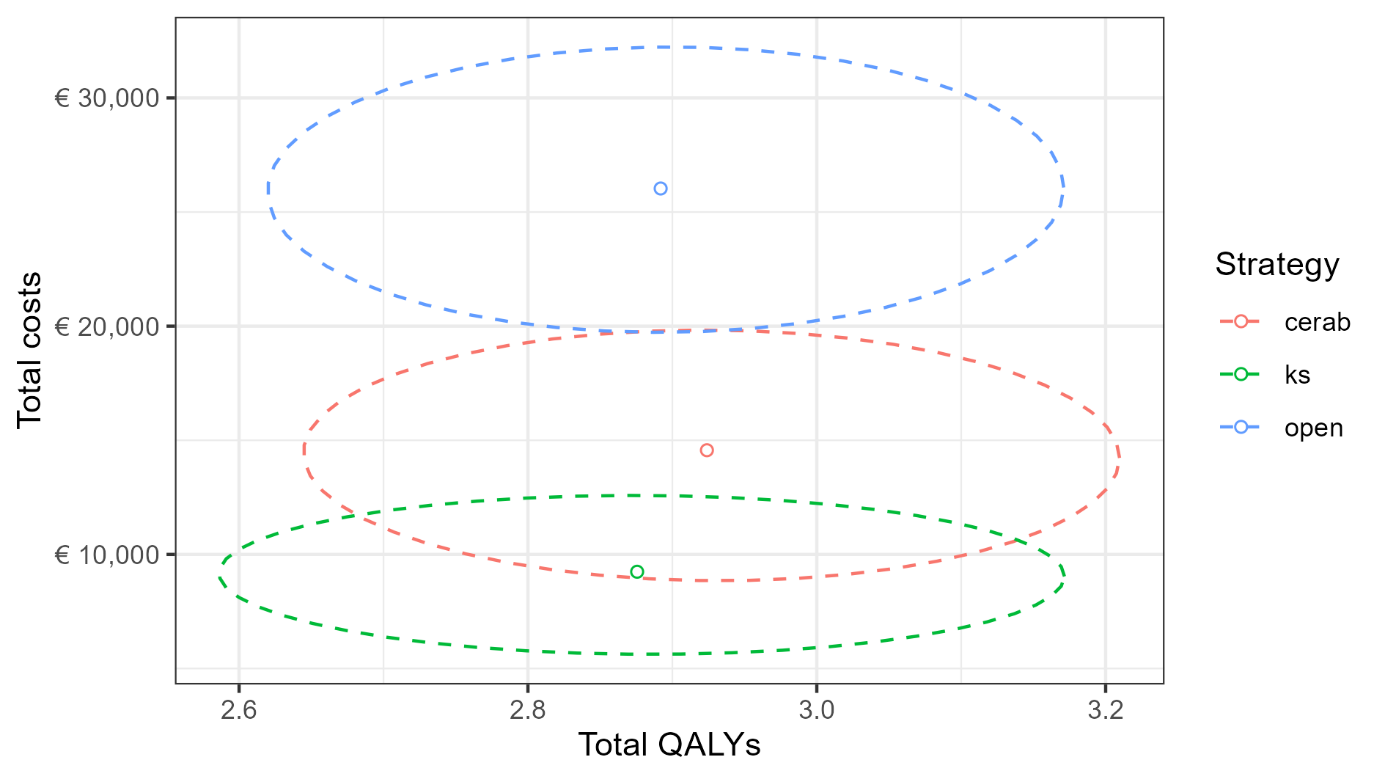


*Figure: Total costs and quality-adjusted life years (QALYs) per individual per strategy*

## Sensitivity analysis shorter length of stay at the ICU

The Table below presents the results of the sensitivity analysis assuming a length of stay at the ICU after an OSR of two days. This analysis results in lower incremental costs of OSR versus KS but does not influence the conclusion of the evaluation.

Table: Sensitivity analysis results - shorter ICU stay after OSR

| Strategy | Mean LY (95%CI) | Probability reintervention (95%CI) | Mean QALY (95%CI) | Mean costs (95%CI) | Incremental QALY | Incremental costs | ICER |
| --- | --- | --- | --- | --- | --- | --- | --- |
| KS | 4.32 (4.24 - 4.35) | 29 % (17% - 46%) | 2.88 (2.6 - 3.13) | € 9,178 (€ 6,560 - € 13,106)* | NA | € NA | Start comparison |
| OSR | 4.23 (4.19 - 4.26) | 6 % (1% - 15%) | 2.90 (2.63 - 3.14)* | € 16,524 (€ 12,877 - € 20,886) | 0.02 | € 7,346 | Dominated |
| CERAB | 4.32 (4.24 - 4.35) | 17 % (11% - 27%) | 2.92 (2.65 - 3.17) | € 14,502 (€ 10,419 - € 20,722)* | 0.05 | € 5,324 | € 110,201 |
| ** These results are not exactly equal to the base-case results since failure of KS or CERAB can lead to a conversion to OSR (hence the differences in costs for KS and CERAB compared to the base-case) and because the length of being at the ICU affects the quality of life of individuals (hence the small difference in QALYs in the OSR strategy).* | | | | | | | |

## CERAB versus KS: probabilistic one-way sensitivity analysis

This figure shows the results of the probabilistic one-way sensitivity analyses of CERAB versus KS, using 1,500 iterations. For clarity, only the 10 most influential parameters were included in this figure. On the x-axis of this figure, the incremental net monetary benefit is plotted*. On the y-axis, the name of the parameters are provided. Based on these results, the most influential parameters on the comparison of CERAB versus KS are the number of stents needed for a CERAB surgery and the parameters of the survival models used to estimate the probabilities of reintervention in both CERAB and KS strategies.

** iNMB = iQALY * WTP – iCosts
where
iNMB = incremental net benefit
iQALY = difference in QALY between CERAB and KS
iCosts = difference in costs between CERAB and KS*


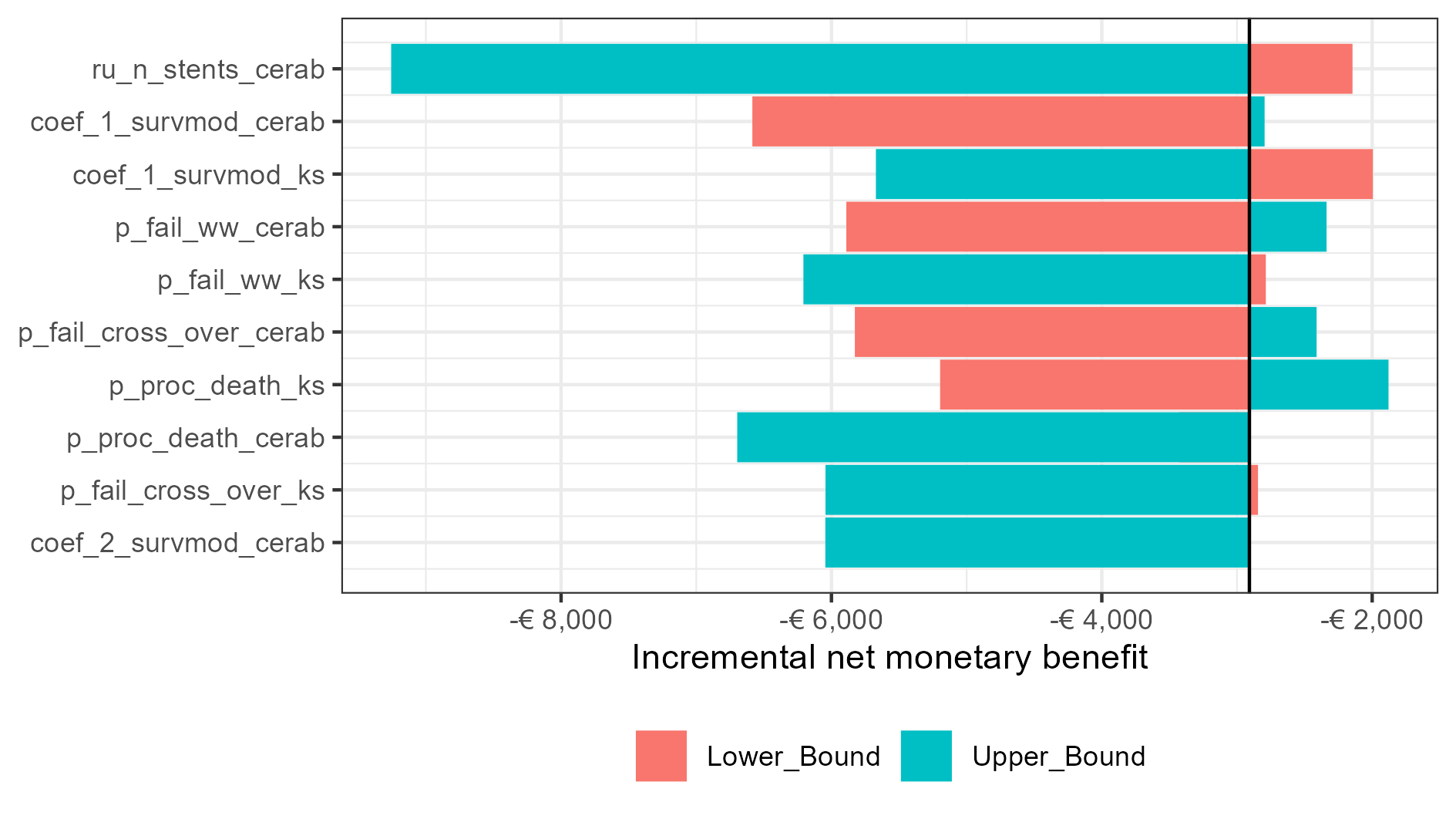


Explanation of the abbreviations of model input parameters:

- ru_n_stents_cerab = number of stents used during a CERAB surgery
- coef_1_survmod_cerab = first coefficient of the survival model used to estimate the probability of reintervention in the CERAB strategy (meanlog of shape depending on survival model)
- coef_1_survmod_KS = first coefficient of the survival model used to estimate the probability of reintervention in the KS strategy (meanlog of shape depending on survival model)
- p_fail_ww_cerab = probability of performing “watchful waiting” treatment strategy in case of failure of placement of CERAB
- p_fail_ww_ks = probability of performing “watchful waiting” treatment strategy in case of failure of placement of KS
- p_fail_cross_cerab = probability of performing a “cross over” treatment strategy in case of failure of placement of CERAB
- p_proc_death_ks = probability of procedure-related death in the KS strategy
- p_proc_death_cerab = probability of procedure-related death in the CERAB strategy
- p_fail_cross_ks = probability of performing a “cross over” treatment strategy in case of failure of placement of KS
- coef_2_survmod_cerab = second coefficient of the survival model used to estimate the probability of reintervention in the CERAB strategy (sdlog of rate depending on survival model)

## OSR versus KS: probabilistic one-way sensitivity analysis

This figure shows the results of the probabilistic one-way sensitivity analyses of OSR versus KS, using 1,500 iterations. For clarity, only the 10 most influential parameters were included in this figure. On the x-axis of this figure, the incremental net monetary benefit is plotted*. On the y-axis, the name of the parameters are provided. Based on these results, the three most influential parameters on the comparison of OSR versus KS are: the number of ICU days after an OSR, the number of minutes needed to perform an OSR, and the number of minutes needed to perform the KS surgery.

** iNMB = iQALY * WTP – iCosts
where
iNMB = incremental net benefit
iQALY = difference in QALY between CERAB and KS
iCosts = difference in costs between CERAB and KS*


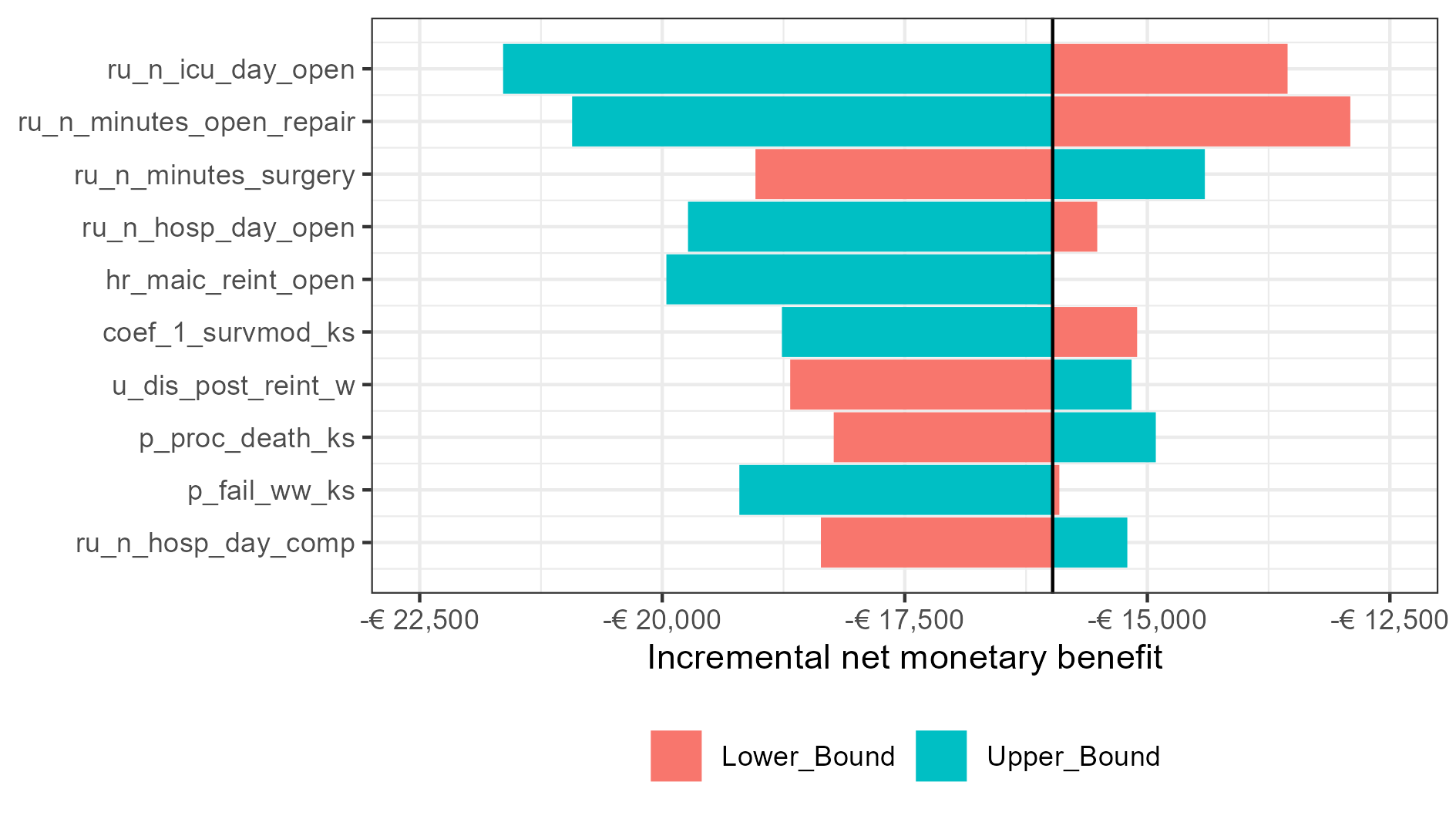


Explanation of the abbreviations of model input parameters:

- ru_n_icu_day_open = number of intensive care unit day in the OSR strategy after a OSR
- ru_n_minutes_open_repair = duration in minutes of a OSR
- ru_n_minutes_surgery = duration in minutes of a KS surgery
- ru_n_hosp_day_open = number of hospital day in the OSR strategy after a OSR
- hr_maic_reint_open = hazard ratio of OSR versus KS (and CERAB) obtained from the matching-adjusted indirect treatment comparison
- coef_1_survmod_KS = first coefficient of the survival model used to estimate the probability of reintervention in the KS strategy (meanlog of shape depending on survival model)
- u_dis_post_reint_w = disutility value after a reintervention in women
- p_proc_death_ks = probability of procedure-related death in the KS strategy
- p_fail_ww_ks = probability of performing “watchful waiting” treatment strategy in case of failure of placement of KS
- ru_n_hosp_day_comp = number of hospital day in the KS strategy after a KS surgery if a complication occurred after the surgery

## Subgroup analyses: probability of reintervention

The following graphs show the cumulative probability of reintervention obtained in the different subgroup analyses.


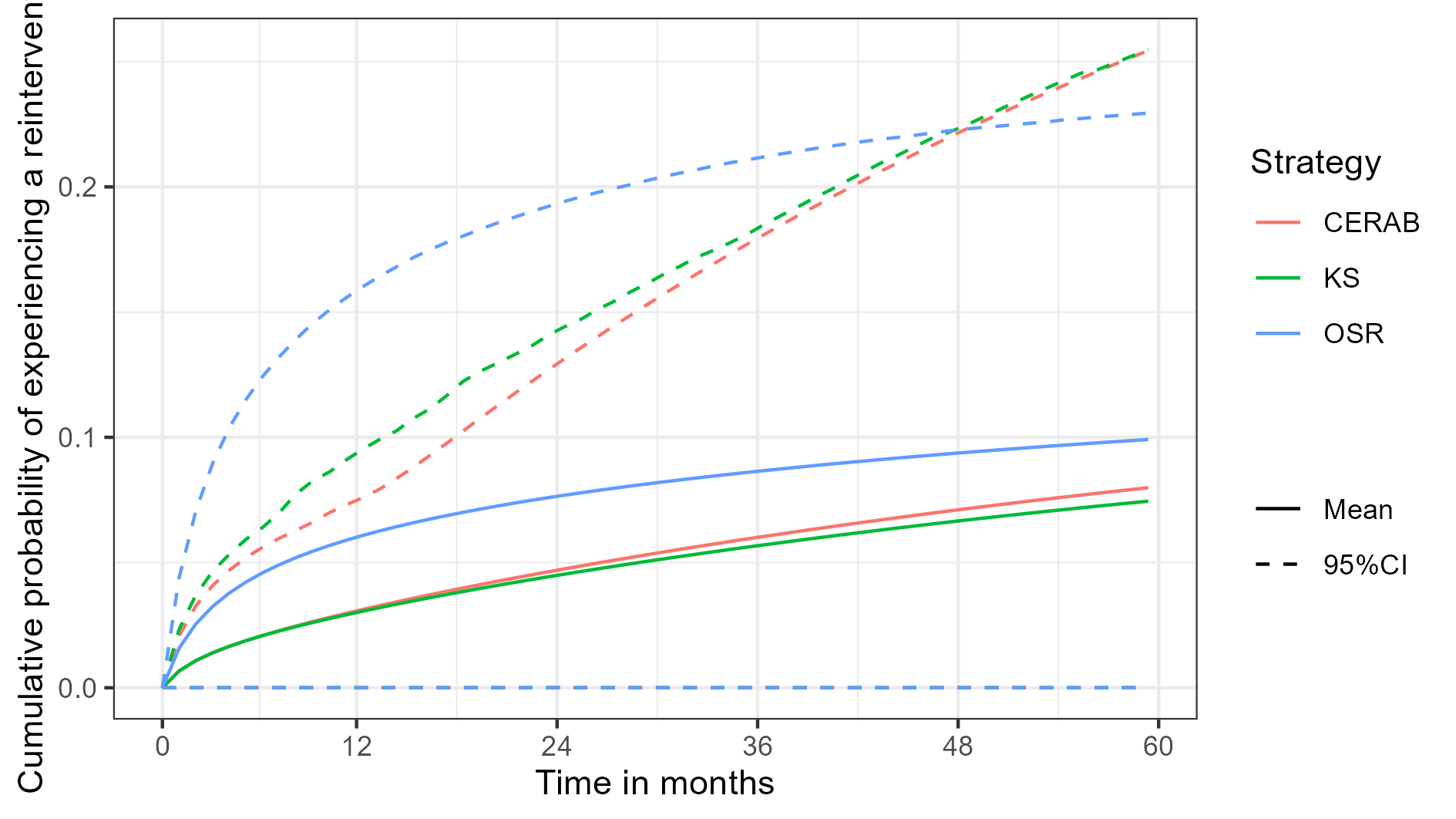


Cumulative probability of reintervention in each stategy - 65+ years old subgroup


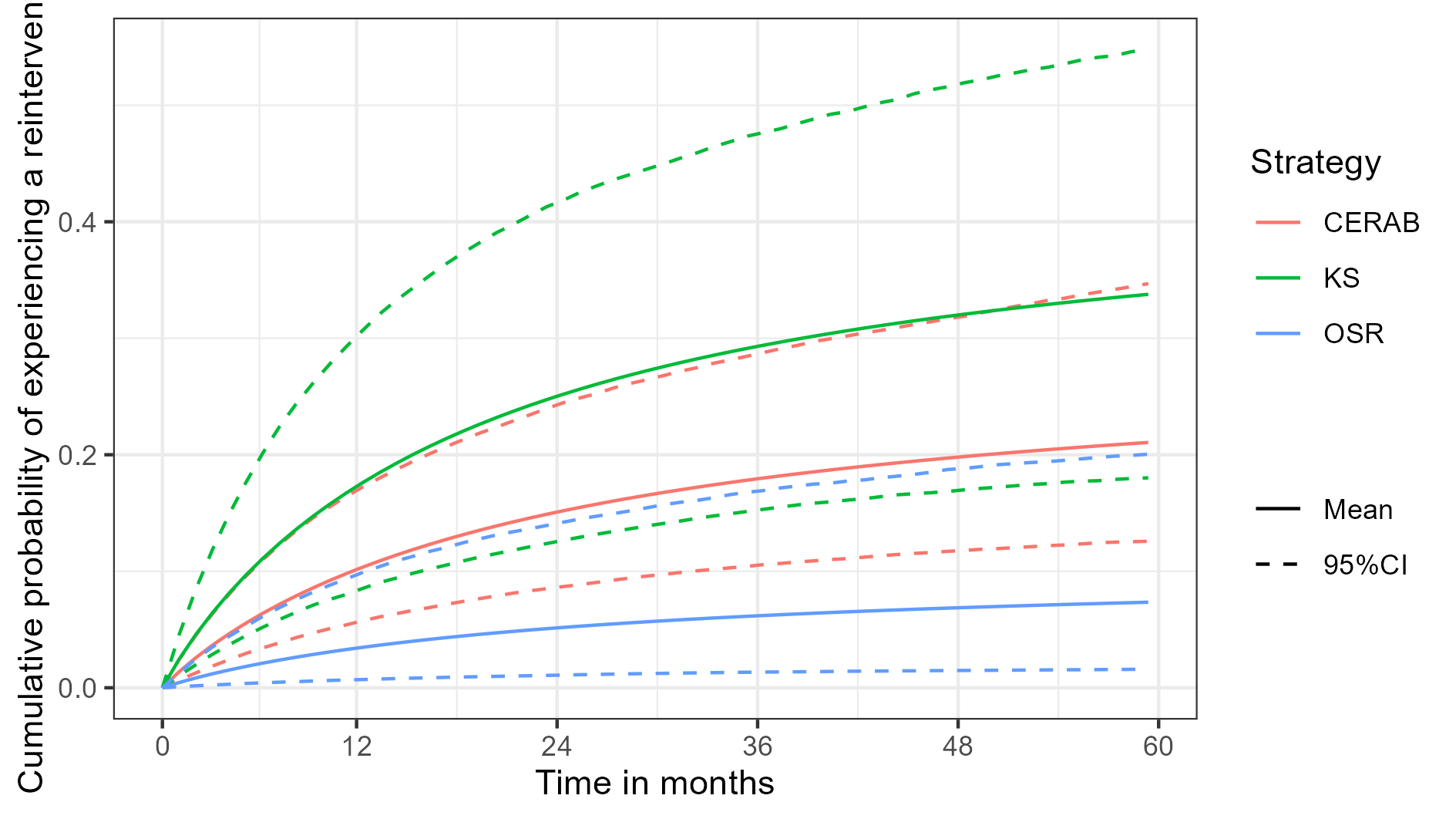


Cumulative probability of reintervention in each stategy - 65- years old subgroup


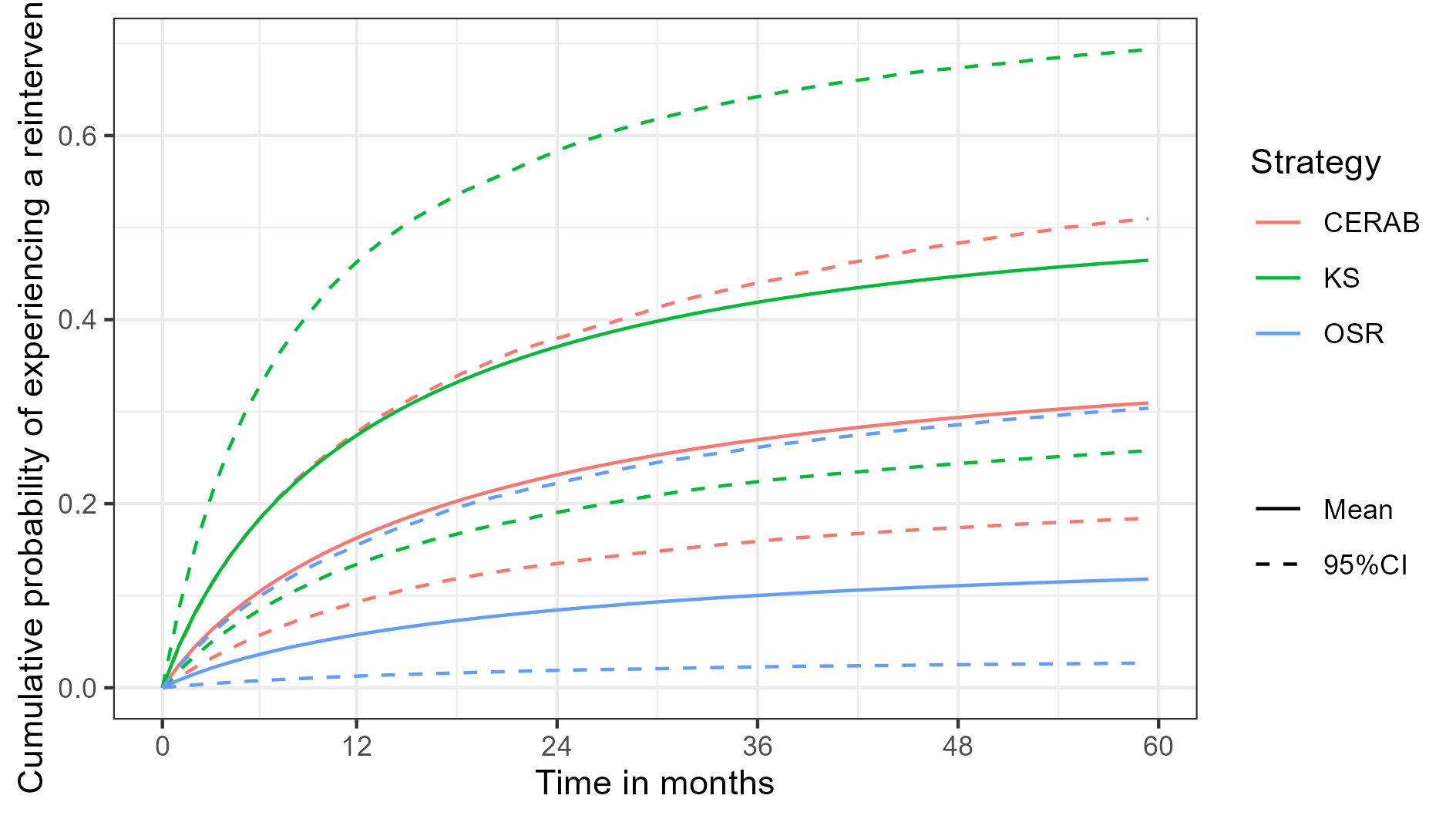


Cumulative probability of reintervention in each stategy - previous reintervention subgroup


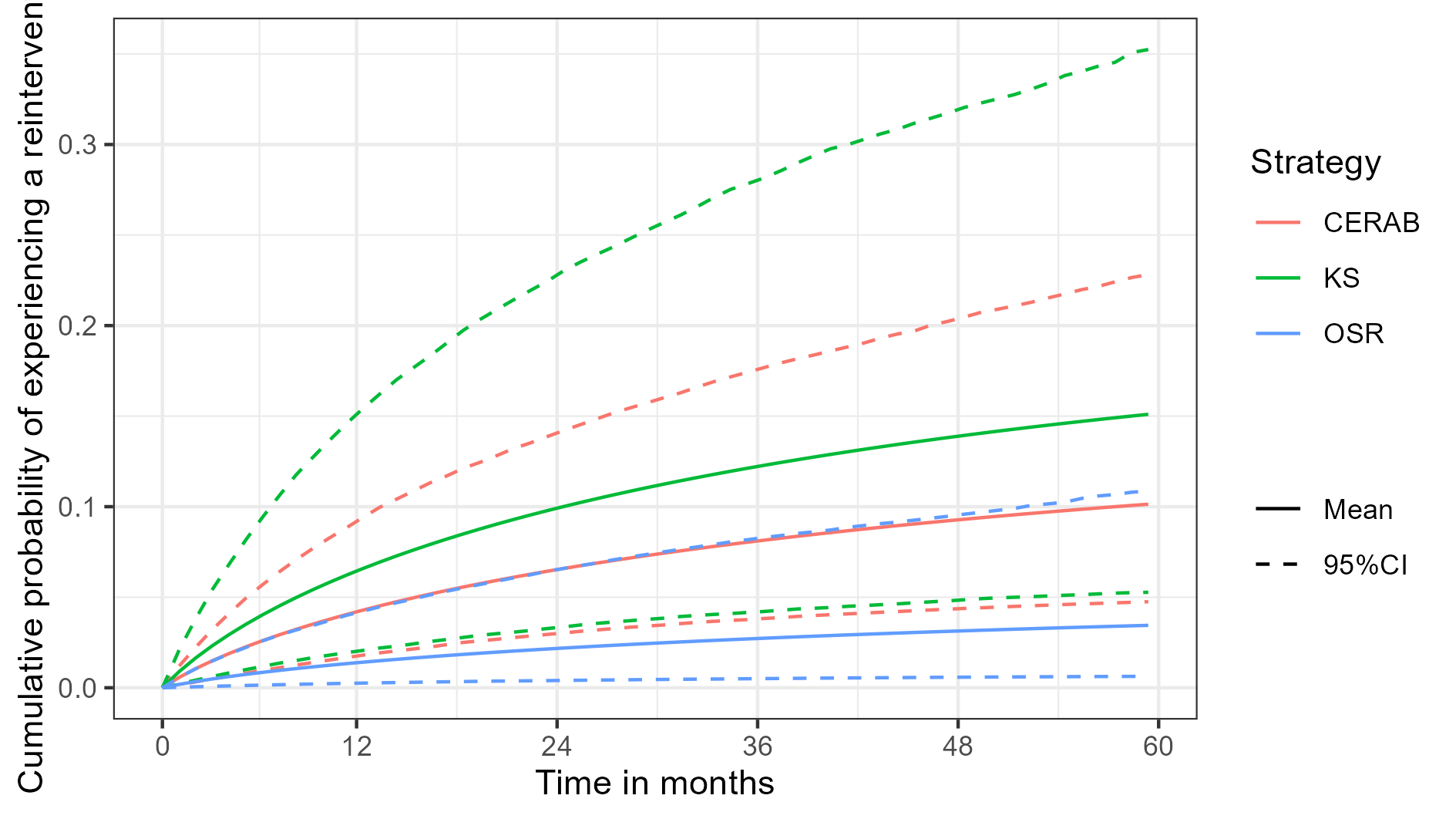


Cumulative probability of reintervention in each stategy - no previous reintervention subgroup

## Convergence incremental costs & QALYs

The Figure below shows the relation between the mean incremental QALYs and costs estimates versus the number of iterations (on the log scale). Based on these figures, we can argue that we performed enough iterations to obtain stable estimates of the mean incremental QALYs and costs.

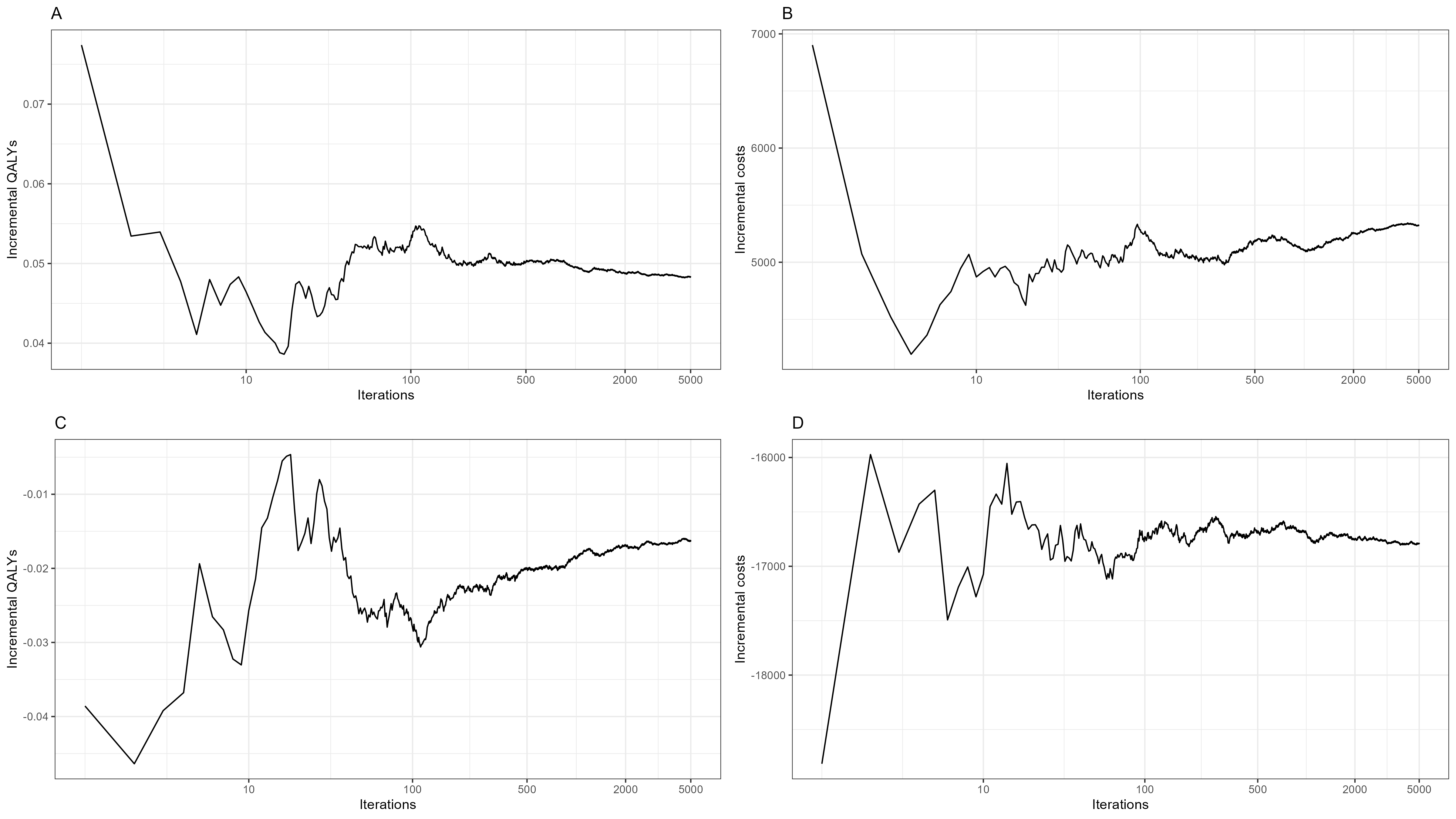
 *Legend: A = convergence plot of incremental QALYs of CERAB versus Kissing Stents, B = convergence plot of incremental costs of CERAB versus Kissing Stents, C = convergence plot of incremental QALYs of open repair versus Kissing Stents, D = convergence plot of incremental costs of open repair versus Kissing Stents*

# R session information

## R version 4.3.2 (2023-10-31 ucrt)
## Platform: x86_64-w64-mingw32/x64 (64-bit)
## Running under: Windows 11 x64 (build 22631)
##
## Matrix products: default
##
##
## locale:
## [1] LC_COLLATE=Dutch_Netherlands.utf8 LC_CTYPE=Dutch_Netherlands.utf8
## [3] LC_MONETARY=Dutch_Netherlands.utf8 LC_NUMERIC=C
## [5] LC_TIME=Dutch_Netherlands.utf8
##
## time zone: Europe/Amsterdam
## tzcode source: internal
##
## attached base packages:
## [1] stats graphics grDevices utils datasets methods base
##
## other attached packages:
## [1] CERAB_0.0.0.9000 testthat_3.2.1
##
## loaded via a namespace (and not attached):
## [1] rgenoud_5.9-0.10 gridExtra_2.3 remotes_2.4.2.1
## [4] writexl_1.4.2 sandwich_3.1-0 rlang_1.1.3
## [7] magrittr_2.0.3 compiler_4.3.2 callr_3.7.3
## [10] vctrs_0.6.5 reshape2_1.4.4 quadprog_1.5-8
## [13] stringr_1.5.1 profvis_0.3.8 pkgconfig_2.0.3
## [16] crayon_1.5.2 fastmap_1.1.1 backports_1.4.1
## [19] ellipsis_0.3.2 labeling_0.4.3 utf8_1.2.4
## [22] promises_1.2.0.1 deSolve_1.40 rmarkdown_2.16
## [25] sessioninfo_1.2.2 tzdb_0.4.0 ps_1.7.6
## [28] purrr_1.0.2 xfun_0.33 cachem_1.0.6
## [31] jsonlite_1.8.8 mstate_0.3.2 highr_0.10
## [34] later_1.3.0 parallel_4.3.2 broom_1.0.5
## [37] prettyunits_1.1.1 R6_2.5.1 stringi_1.7.8
## [40] car_3.1-2 boot_1.3-28.1 pkgload_1.3.4
## [43] brio_1.1.4 numDeriv_2016.8-1.1 iterators_1.0.14
## [46] Rcpp_1.0.9 knitr_1.40 usethis_2.2.2
## [49] zoo_1.8-12 downloader_0.4 readr_2.1.5
## [52] httpuv_1.6.14 Matrix_1.6-5 splines_4.3.2
## [55] tidyselect_1.2.0 rstudioapi_0.14 abind_1.4-5
## [58] yaml_2.3.6 codetools_0.2-19 doParallel_1.0.17
## [61] miniUI_0.1.1.1 processx_3.8.3 pkgbuild_1.3.1
## [64] lattice_0.22-5 tibble_3.2.1 plyr_1.8.9
## [67] shiny_1.8.0 withr_3.0.0 evaluate_0.23
## [70] desc_1.4.3 survival_3.5-7 urlchecker_1.0.1
## [73] muhaz_1.2.6.4 fitdistrplus_1.1-11 pillar_1.9.0
## [76] ggpubr_0.6.0 carData_3.0-5 whisker_0.4.1
## [79] foreach_1.5.2 generics_0.1.3 rprojroot_2.0.4
## [82] truncnorm_1.0-9 hms_1.1.3 ggplot2_3.4.4
## [85] munsell_0.5.0 scales_1.3.0 xtable_1.8-4
## [88] mc2d_0.2.0 glue_1.6.2 flexsurv_2.2.2
## [91] tools_4.3.2 data.table_1.15.0 ggsignif_0.6.4
## [94] fs_1.5.2 mvtnorm_1.2-4 cowplot_1.1.3
## [97] grid_4.3.2 tidyr_1.3.1 devtools_2.4.5
## [100] colorspace_2.1-0 Matching_4.10-14 cli_3.6.2
## [103] fansi_1.0.6 dplyr_1.1.4 gtable_0.3.3
## [106] cbsodataR_1.0.1 rstatix_0.7.2 digest_0.6.34
## [109] farver_2.1.1 htmlwidgets_1.5.4 memoise_2.0.1
## [112] htmltools_0.5.7 lifecycle_1.0.3 statmod_1.5.0
## [115] mime_0.12 MASS_7.3-60.0.1

# References

[1] van Buuren S, Groothuis-Oudshoorn K. mice: Multivariate imputation by chained equations in r. *Journal of Statistical Software*. 2011;45(3):1-67. doi:[10.18637/jss.v045.i03](https://doi.org/10.18637/jss.v045.i03)

[2] Quartagno M, Carpenter J. *jomo: A Package for Multilevel Joint Modelling Multiple Imputation*.; 2022. <https://CRAN.R-project.org/package=jomo>

[3] Groot Jebbink E, Holewijn S, Versluis M, Grimme F, Hinnen JW, Sixt S, et al. Meta-analysis of individual patient data after kissing stent treatment for aortoiliac occlusive disease. *Journal of Endovascular Therapy*. 2019;26(1):31-40.

[4] Hinnen J, Konickx M, Meerwaldt R, Kolkert J, Palen J van der, Huisman A, et al. Long term results of kissing stents in the aortic bifurcation. *Acta chirurgica Belgica*. 2015;115(3):191-197.

[5] Dorigo W, Piffaretti G, Benedetto F, Tarallo A, Castelli P, Spinelli F, et al. A comparison between aortobifemoral bypass and aortoiliac kissing stents in patients with complex aortoiliac obstructive disease. *Journal of Vascular Surgery*. 2017;65(1):99-107.

[6] Phillippo D, Ades T, Dias S, Palmer S, Abrams KR, Welton N. NICE DSU technical support document 18: Methods for population-adjusted indirect comparisons in submissions to NICE. Published online 2016.

[7] Salem M, Hosny MS, Francia F, Sallam M, Saratzis A, Saha P, et al. Management of extensive aorto-iliac disease: A systematic review and meta-analysis of 9319 patients. *CardioVascular and Interventional Radiology*. 2021;44:1518-1535.

[8] Premaratne S, Newman J, Hobbs S, Garnham A, Wall M. Meta-analysis of direct surgical versus endovascular revascularization for aortoiliac occlusive disease. *Journal of Vascular Surgery*. 2020;72(2):726-737.

[9] Rogula B, Lozano-Ortega G, Johnston KM. A method for reconstructing individual patient data from kaplan-meier survival curves that incorporate marked censoring times. *MDM Policy & Practice*. 2022;7(1):23814683221077643.

[10] Saratzis A, Salem M, Sabbagh C, Abisi S, Huasen B, Egun A, et al. Treatment of aortoiliac occlusive disease with the covered endovascular reconstruction of the aortic bifurcation (CERAB) technique: Results of a UK multicenter study. *Journal of Endovascular Therapy*. 2021;28(5):737-745.

[11] de Jonge E. *cbsodataR: Statistics Netherlands (CBS) Open Data API Client*.; 2020. <https://CRAN.R-project.org/package=cbsodataR>

[12] Chalmers N, Walker PT, Belli AM, Thorpe AP, Sidhu PS, Robinson G, et al. Randomized trial of the SMART stent versus balloon angioplasty in long superficial femoral artery lesions: The SUPER study. *Cardiovascular and interventional radiology*. 2013;36(2):353-361.

[13] Vries M de, Ouwendijk R, Kessels AG, Haan MW de, Flobbe K, Hunink MG, et al. Comparison of generic and disease-specific questionnaires for the assessment of quality of life in patients with peripheral arterial disease. *Journal of vascular surgery*. 2005;41(2):261-268.

[14] Versteegh MM, Vermeulen KM, Evers SM, De Wit GA, Prenger R, Stolk EA. Dutch tariff for the five-level version of EQ-5D. *Value in health*. 2016;19(4):343-352.

[15] Van Stel HF, Busschbach JJ, Hunink MM, Buskens E. Impact of secondary cardiovascular events on health status. *Value in Health*. 2012;15(1):175-182.

[16] Tangelder MJ, McDonnel J, Van Busschbach JJ, Buskens E, Algra A, Lawson JA, et al. Quality of life after infrainguinal bypass grafting surgery. *Journal of vascular surgery*. 1999;29(5):913-919.

[17] Bosch JL, Graaf Y van der, Hunink MG. Health-related quality of life after angioplasty and stent placement in patients with iliac artery occlusive disease: Results of a randomized controlled clinical trial. *Circulation*. 1999;99(24):3155-3160.

[18] Mayor J, Branco BC, Chung J, Montero-Baker MF, Kougias P, Mills Sr JL, et al. Outcome comparison between open and endovascular management of TASC II d aortoiliac occlusive disease. *Annals of Vascular Surgery*. 2019;61:65-71.

[19] Tong Y, Khachane A, Ibrahim M, Jacob T, Shiferson A, Almadani M, et al. Open abdominal aortic repair in the current era has more complications for occlusive disease than for aneurysm repair. *Journal of Vascular Surgery*. 2023;77(2):432-439.

[20] Oostenbrink JB, Tangelder MJ, Busschbach JJ, Hout BA van, Buskens E, Algra A, et al. Cost-effectiveness of oral anticoagulants versus aspirin in patients after infrainguinal bypass grafting surgery. *Journal of vascular surgery*. 2001;34(2):254-262.

[21] Schaik TG van, Yeung KK, Verhagen HJ, Bruin JL de, Sambeek MR van, Balm R, et al. Long-term survival and secondary procedures after open or endovascular repair of abdominal aortic aneurysms. *Journal of vascular surgery*. 2017;66(5):1379-1389.

[22] Vreman RA, Geenen JW, Knies S, Mantel-Teeuwisse AK, Leufkens HG, Goettsch WG. The application and implications of novel deterministic sensitivity analysis methods. *Pharmacoeconomics*. 2021;39:1-17.

[23] McCabe C, Paulden M, Awotwe I, Sutton A, Hall P. One-way sensitivity analysis for probabilistic cost-effectiveness analysis: Conditional expected incremental net benefit. *PharmacoEconomics*. 2020;38:135-141.

[24] Wickham H. Testthat: Get started with testing. *The R Journal*. 2011;3:5-10. <https://journal.r-project.org/archive/2011-1/RJournal_2011-1_Wickham.pdf>

[25] Piffaretti G, Fargion AT, Dorigo W, Pulli R, Gattuso A, Bush RL, et al. Outcomes from the multicenter italian registry on primary endovascular treatment of aortoiliac occlusive disease. *Journal of Endovascular Therapy*. 2019;26(5):623-632.

[26] Heijink R, Baal P van, Oppe M, Koolman X, Westert G. Decomposing cross-country differences in quality adjusted life expectancy: The impact of value sets. *Population health metrics*. 2011;9:1-11.
